# Tissue macrophages and interferon-gamma signalling control blood-stage *Plasmodium chabaudi* infections derived from mosquito-transmitted parasites

**DOI:** 10.1016/j.crimmu.2021.07.002

**Published:** 2021-07-30

**Authors:** Katrien Deroost, Christopher Alder, Caroline Hosking, Sarah McLaughlin, Jing-Wen Lin, Matthew D. Lewis, Yolanda Saavedra-Torres, John W.G. Addy, Prisca Levy, Maria Giorgalli, Jean Langhorne

**Affiliations:** The Francis Crick Institute, London, UK

**Keywords:** *Plasmodium chabaudi*, Spleen, Transcriptomics, Macrophages, Red pulp macrophages, IFNγ

## Abstract

Natural infection with *Plasmodium* parasites, the causative agents of malaria, occurs via mosquito vectors. However, most of our knowledge of the immune response to the blood stages of *Plasmodium* is from infections initiated by injection of serially blood-passaged infected red blood cells, resulting in an incomplete life cycle in the mammalian host. Vector transmission of the rodent malaria parasite, *Plasmodium chabaudi chabaudi* AS has been shown to give rise to a more attenuated blood-stage infection in C57Bl/6J mice, when compared to infections initiated with serially blood-passaged *P. chabaudi*-infected red blood cells. In mouse models, the host immune response induced by parasites derived from natural mosquito transmission is likely to more closely resemble the immune responses to *Plasmodium* infections in humans. It is therefore important to determine how the host response differs between the two types of infections.

As the spleen is considered to be a major contributor to the protective host response to *P. chabaudi*, we carried out a comparative transcriptomic analysis of the splenic response to recently mosquito-transmitted and serially blood-passaged parasites in C57Bl/6J mice. The attenuated infection arising from recently mosquito-transmitted parasites is characterised by an earlier and stronger myeloid- and IFNγ-related response. Analyses of spleen lysates from the two infections similarly showed stronger or earlier inflammatory cytokine and chemokine production in the recently mosquito-transmitted blood-stage infections. Furthermore, tissue macrophages, including red pulp macrophages, and IFNγ-signalling in myeloid cells, are required for the early control of *P. chabaudi* recently mosquito-transmitted parasites, thus contributing to the attenuation of mosquito-transmitted infections.

The molecules responsible for this early activation response to recently-transmitted blood-stage parasites in mice would be important to identify, as they may help to elucidate the nature of the initial interactions between blood-stage parasites and the host immune system in naturally transmitted malaria.

## Highlights

• Attenuation of recently transmitted malaria happens at blood stage of infection.
• Stronger or earlier inflammatory cytokine and chemokine production.
• Tissue macrophages, including red pulp macrophages, contribute to attenuation.
• IFNγ-signalling in myeloid cells is required for early control of *P. chabaudi* AS.

## Graphical abstract

**Figure:**
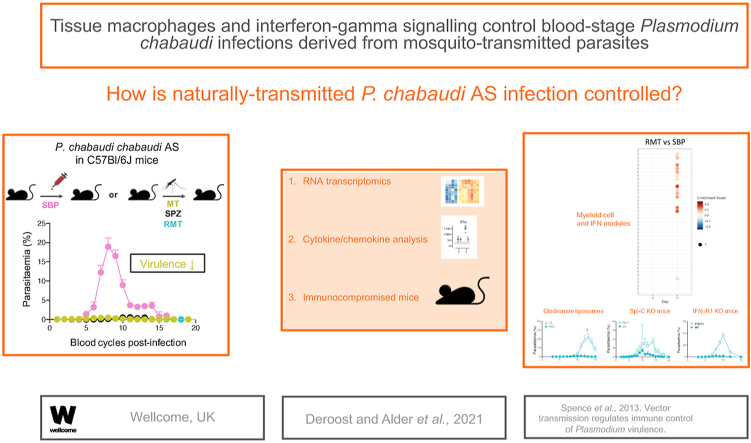

## 1 Introduction

The blood stages of the malaria parasite, *Plasmodium,* in the vertebrate host are able to induce strong immune responses, which can control parasite growth, but also contribute to severe complications of malaria such as severe malarial anaemia, acute respiratory distress and cerebral malaria [reviewed in (Deroost et al., 2016)]. Understanding the nature and regulation of host immune responses to malaria is therefore important for the development of successful intervention therapies.

Most studies designed to determine mechanisms of immunity and immune-regulation in blood-stage *Plasmodium* infections rely on animal models. These typically involve directly injected infected red blood cells (iRBCs) from multiple blood-passaged parasites, bypassing the natural route of transmission and the full life cycle in the mosquito vector and vertebrate host, including the liver stage. While studies in a variety of mouse models have contributed substantially to our understanding of the nature of immune responses in the development of protective immunity and immunopathology [reviewed in (Deroost et al., 2016)], infections carried out in this manner may not completely replicate the natural course of infection in human malaria where parasites are inoculated into the skin following mosquito bite, and first develop in the liver before going into the bloodstream.

We have previously reported that transcriptomes of the blood stages of the rodent malaria parasite, *Plasmodium chabaudi chabaudi* AS obtained from a mosquito-transmitted (MT) infection differ from those of serially blood-passaged parasites (SBP) (Spence et al., 2013). Furthermore, these MT parasites, or parasites that have recently been transmitted through the mosquito (recently mosquito-transmitted, RMT) give rise to an attenuated blood-stage infection with reduced parasitaemia and reduced pathology (less body weight loss, less anaemia and lower plasma liver enzyme levels) (Brugat et al., 2017; Spence et al., 2013). This attenuation of the blood-stage infection is dependent on an interaction with the host (Brugat et al., 2017; Spence et al., 2013). Our findings suggest that interaction between the host and MT parasites leads to a different or enhanced protective host response, compared with an infection with SBP parasites. Since humans contract malaria after being bitten by infected mosquitoes, it is important to determine what features characterise the host immune response to an infection initiated by parasites via the natural route. These features are more likely to reflect the nature of immune responses elicited during natural human malaria infection.

The attenuation of blood-stage infections induced by mosquito transmission suggests that MT parasites induce a different type of host response: an increase in effectiveness, and/or a change in timing of the host response that controls the infection at a lower parasitaemia and with less pathology. There are many reports showing that the bite of a mosquito, or mosquito saliva, alter the magnitude or type of host response, in some cases enhancing a protective response and in others impeding it (Barros et al., 2016; Bizzarro et al., 2013; Depinay et al., 2006; Donovan et al., 2007; Fonseca et al., 2007; Machain-Williams et al., 2012). Similarly, parasites migrating in the skin or the hepatic stages, may influence the early immune response to the blood stages (Belnoue et al., 2008; Ribot et al., 2019). However, our previous work indicates that attenuation of MT *P. chabaudi* infections is due to the host response induced by blood-stage parasites (Brugat et al., 2017; Spence et al., 2013) and not due to responses to the vector or the preceding hepatic infection, as infection initiated with infected red blood cells derived directly from a mosquito-transmitted infection (RMT) are also attenuated. Furthermore, this attenuation is gradually lost upon repeated serial blood passages (Brugat et al., 2017; Spence et al., 2013).

Here we have compared the mouse immune response to an early blood-stage infection of attenuated (RMT or MT) *P. chabaudi chabaudi* AS with that of an infection initiated with SBP *P. chabaudi chabaudi* AS-iRBCs. As the spleen is considered to be the major organ involved in generating immune responses to *Plasmodium* (Chotivanich et al., 2002; Del Portillo et al., 2012; Kumar et al., 1989; Yap and Stevenson, 1994), we performed comparative transcriptomic and cytokine analysis of the splenic response and performed functional analysis in immunocompromised mice. We show that an early and increased tissue-resident myeloid cell response and IFNγ are important for the attenuation of MT/RMT *P. chabaudi* infections.

## 2 Materials and methods

### 2.1 Mice

C57BL/6J, C57BL/6 *Rag1*^tm1Mom^ (Spanopoulou et al., 1994), *Ighm*^*tm1Cgn*^ [μMT (Kitamura et al., 1991),], *Ifnγr1*^*tm1Agt*^ (Huang et al., 1993), *Ccr2*^*tm1Mae*^ (Boring et al., 1997), 129 S/J, 129SvEv and 129SvEv *Spic*^*tm1Kmm*^ (Kohyama et al., 2009) were bred in the specific pathogen-free facilities of the Francis Crick Institute, London, UK. *Ifnγr2*^*flox/flox*^ (*Ifnγr2*^*fl/fl*^) mice (Lee et al., 2015) were crossed to C57BL/6J *LysM*^*cre*^ (Clausen et al., 1999), *CD11c*^*cre*^ *(**Caton* et al.*, 2007**)*, *CD19*^*cre*^ (Rickert et al., 1997), or *CD4*^*cre*^ *(**Roers* et al.*, 2004**)* mice and were also bred and housed at the Francis Crick Institute. Mice which were heterozygous for the *cre* locus (*cre*^*+/-*^) and homozygous for *Ifnγr2*^*fl/fl*^ were used as the experimental group alongside littermate control mice (either wild-type *cre* mice (*cre*^*−/−*^) homozygous for *ifnγr2*^fl/fl^, or heterozygous *cre*^+/-^ mice without the *Ifnγr2*^*fl/fl*^). Mice were used between 6 and 14 weeks of age. All protocols for breeding and experiments were carried out in accordance with the UK Animals (Scientific Procedures) Act 1986 (Home Office licence 70/8326 and PADD88D48) and were approved by The Francis Crick Institute Ethical Committee.

### 2.2 Parasites and infections

A cloned line of the *Plasmodium chabaudi chabaudi* AS strain originally obtained from David Walliker, University of Edinburgh, UK was passaged through mice by injection of iRBCs and cryopreserved. For infections with *P. chabaudi*, mice were housed in reverse light conditions (dark from 7am to 7pm, light from 7pm till 7am). SBP infections were initiated by intraperitoneal (i.p.) injection of 10^5^ SBP-iRBCs. Mosquito transmission (MT) of *P. chabaudi* was carried out using 20 infected *Anopheles stephensi* strain SD500 mosquitoes as described (Spence et al., 2013). For infections using recently mosquito-transmitted (RMT) *P. chabaudi*, donor mice were infected via mosquito bite to initiate a blood-stage infection. iRBCs (10^5^) from these donor mice were then injected i.p. into the experimental mice to initiate an RMT blood-stage infection. Mice were also infected by intravenous (i.v.) injection of 100 sporozoites (SPZs) isolated from the salivary glands of infected mosquitoes as described (Nahrendorf et al., 2015).

The courses of blood-stage infections were monitored by Giemsa-stained thin blood films or via flow cytometry (CellStream, Luminex), see below, and enumerated as the percentage of iRBCs (parasitaemia). The limit of detection was 0.01 % (Giemsa)-0.05 % (flow cytometry) infected erythrocytes.

Following *P. chabaudi* infections initiated by SPZs or MT, there is a 48h pre-erythrocytic cycle before parasites enter the blood stream. Therefore, the blood-stage infections/parasitaemias here are shown as blood cycles (approximately 24h per cycle) post-infection to enable direct comparison of the courses of infections between directly blood-transmitted and MT- or SPZ-initiated infections, as described (Brugat et al., 2017; Nahrendorf et al., 2015). Mosquito bites and injection of SPZs were performed on day −2, whereas RMT- and SBP-iRBC infections were carried out on day 0.

### 2.3 Determination of parasitaemia via flow cytometry

Parasitaemia was determined by flow cytometry. The protocol of stepwise DNA and RNA staining was optimised from https://nanopdf.com/download/hoechst33342-and-pyronin-y-staining-for-g0-g1-separation_pdf, and used to determine parasitaemia as follows. When parasites were in the trophozoite stage, 1 uL of tail blood was collected in 20 uL of saline-heparin (25 U/mL, Wockhardt) in U-shaped 96-well plates and either stored at 4 °C to be stained later that day or stained immediately in 200 uL Hoechst 33342 (10 ug/mL in PBS supplemented with 2 % fetal bovine serum (FBS) and 3 mM EDTA, ThermoFisher) for 15 min at 37 °C to stain for DNA. After washing twice in PBS/FBS/EDTA, cells were resuspended in 250 uL Pyronin Y (1.5 uM in PBS/FBS/EDTA, Abcam) to stain for RNA and acquired by flow cytometry on a CellStream plate reader (Luminex). A total of 100,000 single RBCs (selected based on their FCS/SSC and FSC/AspectRatio FSC) were acquired for each sample and parasitaemia (% iRBCs) was determined as the % RBCs that contained parasites (DNA^+^RNA^+^ double positive cells).

### 2.4 Splenectomy

Female C57BL/6J mice aged 6–8 weeks were anaesthetised with Isoflurane (IsoFlo® 100 % w/w Inhalation Vapour). For maintenance of anaesthesia, a flow of 150 ml/min of 2–3% Isoflurane in air was delivered by Somnosuite Small Animal Anaesthetic System (Kent Scientific Corporation). Body temperature during anaesthesia was maintained by the use of a feedback-regulated homeothermic blanket (DC Temperature Controller, Stoelting). After induction of anaesthesia, animals received a subcutaneous injection of Buprenorphine (Vetergesic® 0.3 mg/mL solution for injection, 0.1 mg/kg) and Meloxicam (Metacam® 5 mg/ml solution for injection, 10 mg/kg) as pre-emptive analgesia. The abdominal muscle was incised with surgical scissors, and the spleen was then exteriorised and excised by the use of a cauteriser. Sham-splenectomised mice were used as controls, whereby the spleen was not exteriorised, but both the skin and the abdominal muscle were incised. Before suturing the abdominal wall, 0.5 mL of warm sterile saline solution (0.9 % NaCl) was administered intra-abdominally in both splenectomised and sham groups. The abdominal muscle was sutured with absorbable suture and the skin with non-absorbable suture. Emla™ cream 5 % (Lidocaine/prilocaine, Aspen) was applied topically on the surgical wound as topical anaesthetic. Skin sutures were removed 7–10 days post-surgery.

### 2.5 RNA isolation from spleen

Female C57BL/6J mice between 8 and 11 weeks old were infected with 10^5^ SBP or RMT *P. chabaudi*-iRBCs i.p.. At 1, 2, 3, 4 and 6 days post-infection, mice were euthanised, and spleens removed and homogenised in Tri Reagent (Sigma-Aldrich) with a gentleMACS™ Dissociator (Miltenyi Biotec). Spleens from naïve mice of the same age were also collected and used as controls. Samples were snap frozen on dry ice and stored at −80 °C until RNA isolation. Total spleen RNA was extracted using the RiboPure™ RNA extraction kit (Invitrogen, ThermoFisher) according to the manufacturer's instructions. The quantity and quality of the RNA samples was verified using NanoDrop 1000 Spectrophotometer (ThermoFisher Scientific) and Agilent 2100 Bioanalyzer Instrument (Agilent Technologies, Inc.), respectively.

### 2.6 Library prep and sequencing

1 ug of RNA was used for cDNA library preparation using the KAPA RNA Hyperprep with Riboerase kit (Roche). Libraries were normalised, pooled and split across 6 lanes on an Illumina HiSeq4000 sequencer yielding 100 bp paired-end (PE) reads at a depth of 25 million reads.

### 2.7 RNA-seq data analysis

Sample FastQ files from different lanes were not merged until after a preliminary QC using FastQC (Babraham Bioinformatics) and MultiQC (Ewels et al., 2016). Cutadapt (Martin, 2011) v1.18 (parameters: e 0.1, −0 1, -q 10, -m 25) was used to remove adapter sequences and filter reads below 25 bp long. Transcripts were aligned to the *Mus musculus* reference genome GRCm38 v86 using STAR (Dobin et al., 2013) v2.5.2 within RSEM (Li and Dewey, 2011) v1.3.0 (parameters: rsem-calculate-expression, --star, --estimate-rpsd, --paired-end, --seed 1, --star-output-genome-bam). Samtools (Li et al., 2009) v1.3.0 was used to sort and index BAM output files. Count files were imported into the bioconductor package DESeq2 (Love et al., 2014) v1.26 in R v3.6.3 using tximport (Soneson et al., 2015) v1.14. DESeq2 was used to normalise raw count files to adjust for library size and artefacts and to perform variance stabilisation transformation to obtain normalised log_2_ expression values. Genes with counts < 10 in less than 2 samples were filtered out for downstream analysis. Principal component analysis was performed using the top 1000 variable genes within the dataset. Sample correlation was performed on dataset using these top 1000 genes to identify outlier samples (Supplementary Fig. S1). These samples were investigated by microscopy and found to be uninfected. These samples were excluded from downstream analysis. DESeq2 was used for Pairwise comparison and Likelihood-ratio testing in order to determine differentially expressed genes, and only protein-encoding genes were considered. For Pairwise comparisons, each condition and timepoint group was compared against the naïve control using the packages Wald test and false discovery rate (FDR) and *p*-values were corrected for multiple testing using the Benjamini-Hochberg (Lydon et al., 1985) method. Genes with a log_2_ fold change > 1 or < −1 and FDR *p*-value < 0.05 were considered significant. For the LogRatio Test (LRT analysis), the naïve samples were removed from the analysis and a full model (~delivery + day + delivery:day) and reduced model (~delivery + day) was used. In this analysis, genes with an FDR < 0.05 were considered significant. Heatmaps were created using the Bioconductor package ComplexHeatmap v2.20 and other visualisation were performed using R ggplot2 package (Wickham, 2009) v3.3.2.

### 2.8 Modular analysis

Modules, or gene sets, were taken from Singhania et al. (2019) investigating global transcriptional signatures of immune responses to a variety of pathogens, in both blood and lung in which we used the blood modules for analysis. In this paper, the transcriptomes of mouse blood taken at various times in a number of different infections or inflammatory conditions were compared creating a weighted gene correlation network matrix (WGCNA) to identify genes co-regulated together to create the modules and categorise them into known biological functions using various pathway analysis methods (Metacore, Ingenuity pathway (IPA) and Gene ontology (GO) enrichment) to describe the general function of the genes within each module. Modular enrichment analysis was performed using Quantitative set analysis for gene expression (QuSAGE) (Yaari et al., 2013) using the Bioconductor package qusage v2.20. This analysis was done on both routes of infections against the naïve controls, to identify over or under abundant modules for each and then against themselves, to identify any modules significant against each other. The QuSAGE parameter n.points was increased to 2^18^ to account for small sample sizes within groups and to improve accuracy. Modules with an FDR *p*-value < 0.1 were considered significant. For the comparison of infection vs control, the size of the bubbles indicates the proportion of genes within the module to be found differentially expressed by pairwise comparison against the total number of genes within the module. Visualisation was performed using R package ggplot2.

### 2.9 Data and code availability

The RNA-seq data were deposited at the Gene Expression Omnibus database, accession number (GSE169752) and the scripts for data analysis are available at Github link (https://github.com/CJAlder92/EKD11).

### 2.10 Detection of cytokines and chemokines in spleen lysates

Female C57Bl/6J mice aged 8–10 weeks were infected with RMT or SBP *P. chabaudi*-iRBCs. Spleens were excised from mice at day 1, 3, 5, and 7 of the blood-stage infection and from uninfected mice. Spleens were homogenised in RIPA lysis buffer (Sigma-Aldrich) containing cOmplete™, EDTA-free Protease Inhibitor Cocktails (Roche) at 100 mg spleen/1 mL lysis buffer with a POLYTRON homogenizer (Kinematica) on ice, centrifuged at 3,000×*g* for 10 min at 4 °C, and the lysates collected. After two further centrifugation steps at 13,300×*g* for 10 min at 4 °C, lysates were stored at −80 °C till further analysis. Protein content in the supernatants was quantified by Pierce BCA protein assay (Thermo Scientific) as per the manufacturer's instructions. Spleen lysates were analysed with a Mouse Cytokine Array/Chemokine Array 32-Plex (MD31, Eve Technologies, Canada). Five cytokines were omitted from the analysis as their pg/ml were out of range below the 4 or 5 parameter Logistic standard curve.

### 2.11 *In vivo* depletion of myeloid cells

Myeloid cells were depleted via i.v. injection of 200 uL clodronate liposomes as described by the manufacturer (clodronateliposomes.org) starting two days before infection (day −2 for RMT and SBP infections, day -4 for infections initiated via mosquito bite) and subsequently at three days intervals until end of experiment or until mice were culled in line with humane endpoints. In an initial experiment, empty liposomes were used as a control, but as these followed a similar course of infection as wild-type untreated mice, we used wild-type untreated mice as controls in a second experiment. Efficacy of the depletion of myeloid cells was established by flow cytometry, see below.

### 2.12 Flow cytometry

Spleens were excised from mice, passed through a 70 μm cell strainer (Falcon) and washed with complete Iscove's Modified Dulbecco's Medium [IMDM supplemented with 10 % FBS Gold (PAA Laboratories, GE Healthcare), 2 mM L-glutamine, 0.5 mM sodium pyruvate, 100 U penicillin, 100 mg streptomycin, 6 mM Hepes buffer, 50 mM β-ME (all from Gibco, Invitrogen), and 3 mM EDTA] to obtain single cell suspensions. RBCs were lysed for 1 min in RBC lysis buffer (Sigma), washed and passed through a 40 μm cell strainer (Falcon). Viable cells were counted on a haemocytometer with trypan blue exclusion. Cells were then resuspended in PBS containing 2 % FBS and 3 mM EDTA (staining buffer) and seeded in 96-well V-bottom plates and incubated with anti-mouse CD16/CD32 (Fc block, BD Biosciences). After washing once in staining buffer, cells were stained with Live/Dead™ Fixable Blue Dead Cell Stain kit (ThermoFisher) or Zombie UV™ Fixable Viability Kit (Biolegend) and different combinations of the following fluorochrome-labelled antibodies (Biolegend unless otherwise specified) in staining buffer or Brilliant Stain Buffer (BD Biosciences): anti-CD3e (PE), anti-CD8α (BB515, BD Biosciences), anti-CD4 (BUV805, BD Biosciences), anti-NK1.1 (BV711 or PE), anti-γδTCR (APC), anti-CD19 (PE), anti-Ter119 (PE), anti-F4/80 (APC/Cy7), anti-CD11b (PE/dazzle 594), anti-CD169 (BV605 or PE/Cy7), anti-CCR2 (BV421), anti-MHC class II (I-A/I-E, BV510 or AF488), anti-CD11c (BV785 or biotin/SA-BV570), anti-Ly6C (BV711 or BV785), anti-Ly6G (BUV395, BD Biosciences), and anti-MARCO (APC, R&D Systems). Cells were washed twice with staining buffer and either acquired immediately or fixed with 2 % formaldehyde (Alfa Aesar) and stored in staining buffer at 4 **°**C until acquisition**.**

For analysis of myeloid cells, spleens were first cut into small pieces and incubated in Liberase TL (Roche) in complete IMDM without FBS while rotating for 40 min at 37 °C for enzymatic digestion, before passing the cell suspension through a 70 μm cell strainer.

For intracellular staining of IFNγ, cells were incubated for 3 h in complete IMDM, containing Brefeldin A (10 mg/mL; Sigma), and labelled as described above. Cells were fixed in 2 % formaldehyde (Alfa Aesar) and stored in staining buffer overnight at 4 °C, permeabilized in Perm/Wash buffer (BD Biosciences) and incubated in Perm/Wash with PE/Cy7 anti-IFNγ or Isotype (rat IgG1,κ, Biolegend). Cells were washed three times in Perm/Wash buffer, resuspended in staining buffer and acquired immediately. Cell acquisition was performed on a BD LSRFortessa or BD LSRFortessa Symphony (BD Biosciences). Singlets were selected based on FSC-A vs FSC-W and further based on SSC-A vs SSC-W. Dead cells were excluded with the LIVE/DEAD Fixable stain. “Fluorescence minus one” (FMO) controls, in combination with isotype controls, were used to set the thresholds for positive/negative events. Analysis was performed with FlowJo software version 10 (TreeStar).

### 2.13 Statistics

The derivation of the standard error of the mean for percentage parasitaemia was conducted on the log-scale. Peak parasitaemias between groups were compared on log-transformed data with an unpaired *t*-test with Welch's correction. Differences between groups for the flow cytometry data were compared with a nonparametric Mann-Whitney *U* test. Splenic parameters were compared between groups on specific days with a two-way ANOVA and Tukey's multiple comparisons test. Graphs were prepared with Prism 9. Cytokine analysis was conducted on the log-scale using the lm () function in R (R Core Team (2018): https://www.R-project.org/). A nested ANOVA was used on each cytokine comparison, with the day effect nested within RMT (Day 1, 3, 5 and 7), SBP (Day 1, 3, 5 and 7) and Control treatment groups. To test the difference between RMT and SBP treatments at day 5 and 7, respectively, the predictmeans package (https://CRAN.R-project.org/package=predictmeans) was used to extract the associated *p*-value of these differences. Cytokine graphs were made with R.

## 3 Results

### 3.1 Reduced blood-stage parasitaemia after mosquito transmission of *P. chabaudi* AS is not due to a host response in the skin or the liver, but relies on an immune response induced in the spleen

**Fig. 1:**
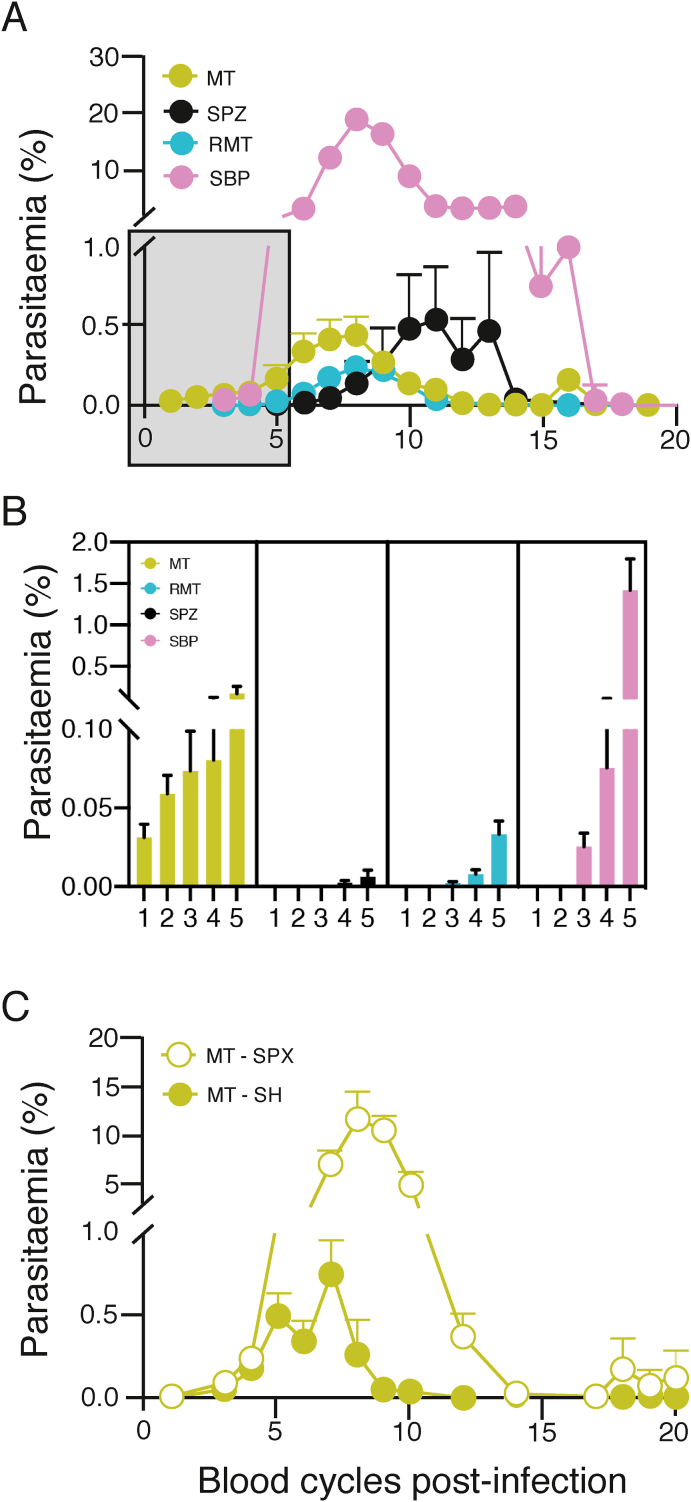
Attenuation is not the result of a host response against pre-erythrocytic stages in the skin or liver and depends on the spleen. **A.** Course of *P. chabaudi* blood-stage infection in female C57Bl/6J mice after mosquito transmission (MT) (green), intravenous (i.v) injection of 100 sporozoites (SPZs) (black), intraperitoneal (i.p.) injection of 10^5^ recently mosquito-transmitted (RMT)-infected red blood cells (iRBCs) (blue) or i.p. injection of 10^5^ serially blood-passaged (SBP)-iRBCs (pink). **B.** Detail of the very early days post-infection depicted in the grey box of Fig. 1A. **C.** Course of an MT *P. chabaudi* blood-stage infection in female C57Bl/6J mice that have been splenectomised (SPX, open symbols) or sham-operated (SH, closed symbols). The graphs in panel **A** and **C** show mean (+/- SEM) of percentage parasitaemia calculated from log-transformed data. Pooled data are shown from individual experiments with n = 42 mice (MT, pooled data from three experiments), n = 13 mice (SPZ, pooled data from two experiments), n = 24 mice (RMT, pooled data from three experiments), and n = 33 mice (SBP, pooled data from six experiments) in panel **A-B** and pooled data from two individual experiments with 10 mice/experiment (SPX) and 7–11 mice/experiment (SH) in panel **C**.

Mosquito transmission of *P. chabaudi* AS results in a reduced blood-stage parasitaemia when compared with infections initiated by SBP-iRBCs (Spence et al., 2013). Here, we compared the courses of infection initiated by: 1) bites of infected mosquitoes (MT infections), 2) intravenous (i.v.) injection of sporozoites (SPZs), thereby omitting the insect bite and presence in the skin, 3) injection of iRBCs obtained from the blood of an MT *P. chabaudi* infection (recently mosquito-transmitted, RMT), thereby obviating all of the pre-erythrocytic stages in the mouse, and 4) injection of SBP *P. chabaudi*-iRBCs. Fig. 1A shows that the attenuation of blood-stage parasitaemia in MT infections was not the result of a host response against the pre-erythrocytic stages of the parasite life cycle in the skin or liver, as i.v. injection of SPZs, or injection of RMT-iRBCs, still resulted in attenuated parasitaemias when compared with a blood-stage infection initiated by iRBCs obtained from an SBP infection, albeit with slightly different kinetics and peak parasitaemia. Infections initiated by the bites of 20 infected mosquitoes (MT) resulted in earlier appearance of parasitaemia; iRBCs were already detectable on thin blood films within 2 days of exiting the liver (i.e. day 2 in the blood), compared with infections initiated by RMT-iRBCs and SBP-iRBCs, which were not detectable until day 3 of the blood cycle (Fig. 1A and B). The numbers of sporozoites developing in the mosquito is not possible to control, therefore the earlier appearance of blood-stage parasites may be because of the greater (and more variable) numbers of SPZs injected during the biting process.

We have shown previously (Brugat et al., 2017; Spence et al., 2013), and verified here (Supplementary Fig. 2A-C), that components of the host immune response such as T cells, but not B cells or antibodies, are required for attenuating the acute stage of MT or RMT blood-stage *P. chabaudi* infections. The spleen is considered to be the major lymphoid organ generating protective immune responses against blood-stage *Plasmodium* and for removing iRBCs (Chotivanich et al., 2002; Del Portillo et al., 2012; Kumar et al., 1989; Yap and Stevenson, 1994), and is therefore likely to be a major source of immune cells needed to control MT or RMT *P. chabaudi* blood-stage infections. To determine the role of the spleen in MT infection, and any role it may have in parasite attenuation, we splenectomised mice and found that acute parasitaemias were substantially higher, and of longer duration, in splenectomised C57Bl/6J mice infected by the bites of infected mosquitoes than parasitaemias in sham-splenectomised (Fig. 1C), or untreated mice (data not shown). This is in accordance with what was shown previously for SBP *P. chabaudi* AS infections (Yap and Stevenson, 1994), indicating that an intact spleen is required for control of *P. chabaudi* parasitaemia no matter the route of infection. Splenectomised mice were able to control the infection to a low parasitaemia level, unlike Rag1 knockout mice lacking B and T cells (Supplementary Fig. S2A), indicating that extrasplenic immune responses, possibly in the lymph nodes or the liver, contribute as well.

Interestingly, despite the much lower peripheral parasitaemias in RMT infections, the kinetics and magnitude of the increase in spleen size were similar between RMT and SBP infections (Supplementary Fig. S3A-C), suggesting that blood-stage parasites that arise from RMT infections may be more effective at activating an early splenic response than SBP-iRBCs. Spleens from infections initiated via mosquito bite started to increase earlier in size than spleens initiated by injection of SPZs or iRBCs, in line with the earlier detection of blood parasites.

As MT- and RMT-initiated infections both attenuate parasitaemia similarly in the absence of a pre-erythrocytic cycle, all subsequent comparisons were carried out with infections initiated with the same dose of RMT- or SBP-iRBCs (10^5^). This also ensures that any responses observed cannot be attributed to those induced by the pre-erythrocytic stages of the *P. chabaudi* parasite*.*

### 3.2 Gene expression in RMT and SBP *P. chabaudi*-infected spleens reveals a small number of differentially expressed genes in the early stage of infection

In order to capture global changes in the splenic host response that might be responsible for the more severe SBP, or the attenuated RMT infections, we performed next generation sequencing on RNA (RNA-seq) isolated from spleens of naïve C57Bl/6J mice, and mice that had been infected with either 10^5^ RMT or SBP *P. chabaud-*iRBCs at several times during the early phase of infection (days 1, 2, 3, 4 and 6 of the blood-stage infection).

Analyses of splenic lymphoid (CD4^+^, CD8^+^ and γδ T cells, natural killer (NK), NK1.1^+^ NKT cells) and myeloid cells (CD11b^+^ Ly6G^+^ neutrophils, MHC class II^hi^ CD11c^hi^ dendritic cells, F4/80^+^ CD11b^−^ red pulp macrophages, CD11b^−^ CD169^+^ marginal metallophilic macrophages, CD11b^−^ MARCO^+^ marginal zone macrophages, or CD11b^+^ Ly6C^+^ CCR2^+/−^ monocytes) by flow cytometry at these timepoints indicated that there were no significant differences in the numbers of any major splenic cell population between RMT- and SBP-initiated infections. (Supplementary Figs. S4 and S5). Therefore, the gene expression profiles should reflect transcriptional changes within the cells rather than selective expansion of particular subpopulations in RMT- or SBP-initiated infections.

**Fig. 2:**
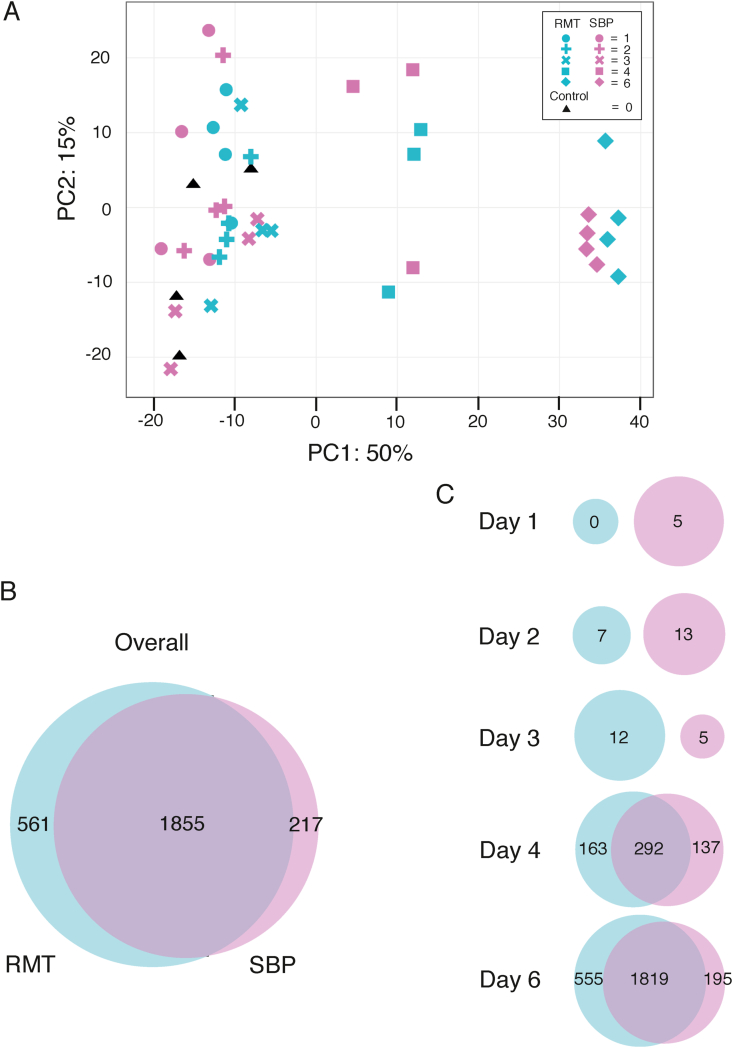
Whole spleen RNAseq of RMT and SBP-infected mice in the first week of infection reveals subtle differences. **A.** Principal component analysis (PCA) of gene expression from the top 1000 variable genes between the transmission types and the timepoints. PC1 and PC2 are used and depict the greatest variation in gene expression between the timepoints and transmissions. Colours indicate the transmission type: pink (SBP), blue (RMT) and black (naïve), whilst symbols indicate days in the blood (0, 1, 2, 3, 4, and 6). Three distinct clusters can be seen with samples clustering by days post-infection. **B.** Venn diagram showing differential gene overlaps of all protein-coding genes between the transmission routes. Differentially expressed genes (DEGs) were identified by comparing the transmission routes against the naïve control (log_2_ fold change > 1 or < −1, FDR < 0.05). **C.** Venn Diagrams showing differences in DEGs identified in RMT and SBP on specific days post-infection.

A principal component analysis (PCA) plot was created using a gene expression matrix of the 1000 most variable genes identified across both modes of infection and all timepoints. Principal component (PC) 1 accounted for 50 % of the variation within the expression matrix and showed distinct clusters for samples from day 4 and day 6, while samples from day 1–3 remained clustered together broadly with samples from naïve mice (Fig. 2A), indicating that not much happens in terms of gene expression compared to uninfected mice until day 4 of the infection. The clusters we see for day 4 and day 6 samples did not separate out RMT from SBP infections, suggesting that differences in gene expression between these infections may be small.

Pairwise comparisons, performed against the naïve control samples, confirmed this and identified a small number of differently expressed genes (DEGs) between the two infection modes. Genes with a log_2_ fold change > 1 or < −1 and a false discovery rate (FDR) < 0.05 were considered differentially expressed. A total of 2416 and 2072 DEGs were identified in RMT and SBP infections respectively, when compared to the uninfected control. All genes identified in each timepoint were used to create a comprehensive list of DEGs for each mode of infection during the time course (Supplementary Tables 1–3). These gene lists were then compared to each other to look for unique DEGs for each mode of infection. The Venn diagram in Fig. 2B shows that a large majority of the genes differentially expressed in each mode of infection over all the time points were shared (1855), with only a small proportion of genes (561 DEGs) unique to RMT infections compared to 217 DEGs unique to SBP. All DEGs shared between RMT and SBP followed the same pattern of up- or down-regulation. When investigating these unique RMT infection DEGs, we see that these genes were also activated in SBP infections, when compared to naïve, but fall below our range of differential expression (Supplementary Table 2). To investigate whether there are temporal differences in gene activation between the two modes of infection, we compared the DEGs identified at each timepoint against each other (Fig. 2C). Day 4 of the blood-stage infection showed the first significant signs of transcriptional activity in both RMT and SBP infections, but the majority of DEGs were found on day 6.

**Fig. 3:**
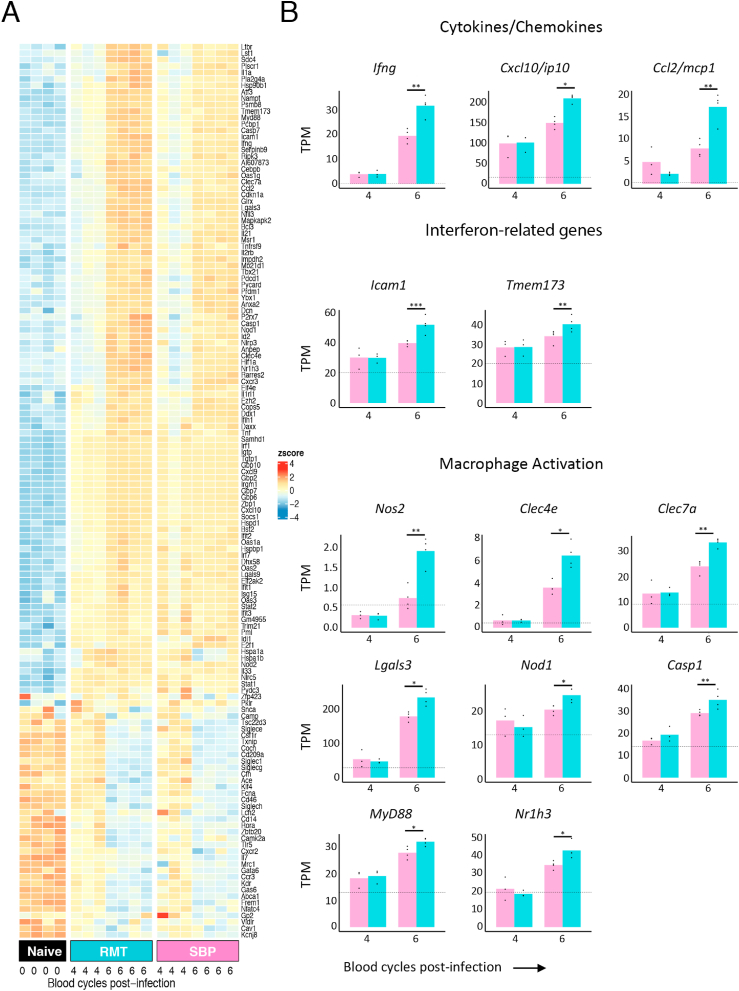
Increased activation of genes associated with interferon pathways and myeloid cell activation in spleens of mice infected with RMT *P. chabaudi* parasites. **A.** Innate DEG Heatmap. Gene expression signature of DEGs identified by comparing infection timepoints against controls. DEGs were then compared to the InnateDB database. Shown are samples from day 4 (n = 3), day 6 (n = 4) from RMT and SBP initiated infections, and naïve samples (n = 4). DEGs compared against naïve samples were selected for the heatmap. Colours represent the Z-score values, which is a measurement of the number of standard deviations a sample value is above or below the mean across all samples for a given gene. **B.** Number of transcripts per million (TPM) of a selection of genes of interest encoding cytokines/chemokines, interferon-related genes, or genes involved in macrophage activation and found as immune-associated genes with the InnateDB in RMT spleens (blue bars) or SBP spleens (pink bars). Lines above groups with asterisks illustrate significances between groups. *, *p* < 0.05; **, *p* < 0.01; ***, *p* < 0.001.

As the attenuation of RMT infections requires a host immune response, we looked for genes associated with the innate immune system in our DEGs using a database of immune associated genes from InnateDB. We found 104 upregulated genes and 36 down-regulated genes in both RMT and SBP infections compared to naïve samples (Fig. 3A). Of the 104 upregulated genes, we found a similar intensity of activation at day 4 for both RMT and SBP infections, however, by day 6, these genes were upregulated to a greater extent in RMT infections. In addition to *ifng* (encoding IFNγ), there were several *ifn*-dependent or *ifn*-induced genes belonging to the type I and/or type II interferon pathway (MacMicking, 2012), including *stat1* (encoding the STAT1 transcription factor), and downstream *ifn*-inducible genes such as *irf1, gbp 6, 7, 10, ifit2, igtp, tgtp1, tbx21* (encoding the transcription factor T-bet), *icam1* and *isg15*. In addition, there were several genes encoding cytokines and chemokines (eg. *ifng, cxcl10/ip10 and ccl2/mcp1*), genes associated with the interferon pathway (e.g. *icam1 and tmem173)* and macrophage-associated genes (eg. *nos2, clec4e, clec7a, lgals3, nod1, casp1, myd88 and nr1h3*). Fig. 3B shows the number of transcripts per million (TPM) of a selection of cytokines, chemokines, interferon-related genes, and indicators of macrophage activation upregulated in RMT infections compared to SBP infections, and all statistically significant genes that are different between RMT and SBP and were found in InnateDB are shown.

This analysis suggests that the innate immune response, particularly genes associated with myeloid responses, the IFNγ pathway, and early events in T cell responses (eg *tbx21*, and *il21*) are expressed more highly in RMT infections than in SBP infections within the first 6 days of a blood-stage infection. This suggests that RMT infections induce a greater macrophage, and IFNγ-dependent response, compared with SBP infections.

### 3.3 Modular analysis and Likelihood Ratio Testing reveals a role for myeloid cells and interferon in attenuation of *P. chabaudi* infection

In order to define more clearly the differences in immune response between RMT and SBP infections, we applied a quantitative modular gene expression approach to look for differences in transcription levels in spleens from both of these infection routes. For this, we used the modules originally described by Singhania et al. (2019).

**Fig. 4:**
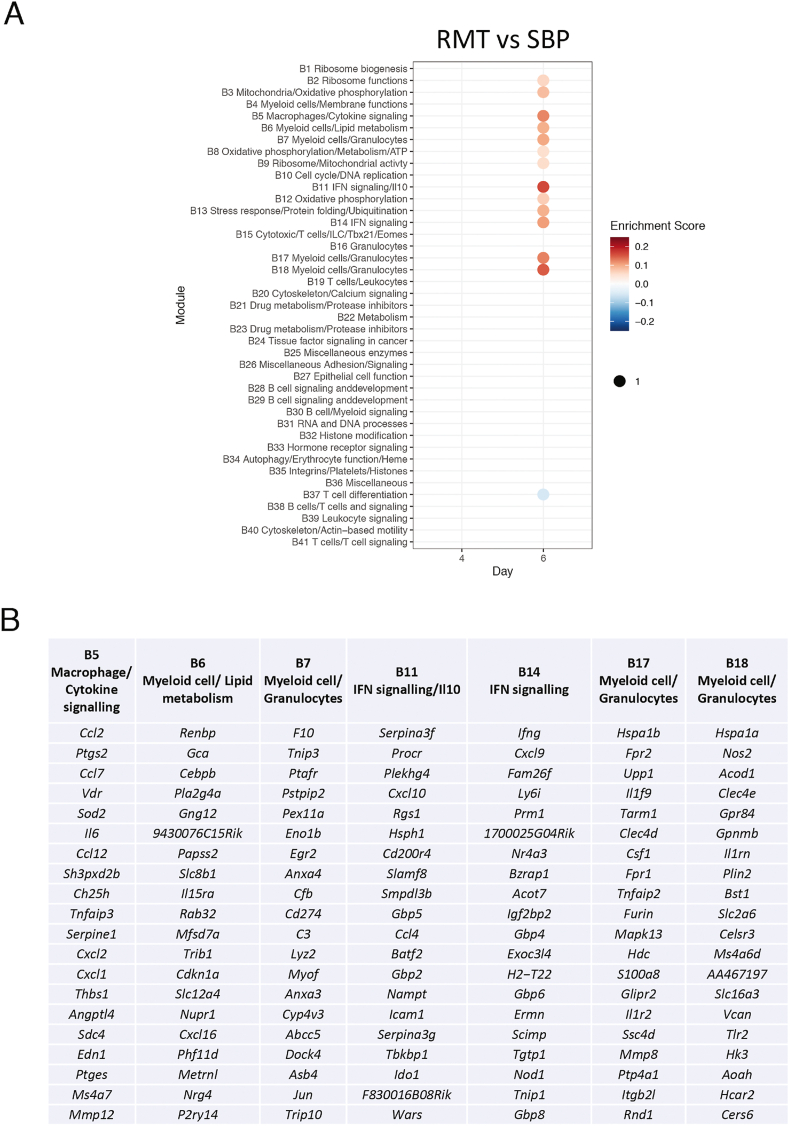
Modular analysis reveals a role for myeloid cells and IFNγ. **A.** Modular transcriptional signatures comparing RMT and SBP samples. Modules taken from Singhania et al. (2019). Module name indicates biological processes associated with the genes within the module. Fold enrichment scores were derived using QuSAGE, with red and blue circles indicating the cumulative over- or under-abundance of all genes within the module. Colour intensity of the dots represents the degree of perturbation, indicated by the colour scale. **B.** Top 20 genes contributing to some of the enriched modules. A list with all genes within each module can be found in Supplementary Table 4.

Direct comparison of gene expression of RMT and SBP infections revealed a number of modules showing greater upregulation in RMT infections only on day 6 of the blood-stage infection (Fig. 4A and Supplementary Table 4). There are modules showing that cellular activity overall is greater in RMT infections; “Ribosome functions” (B9), “Mitochondria/Oxidative phosphorylation” (B3), “Oxidative phosphorylation/Metabolism/ATP” (B8), “Oxidative phosphorylation” (B12), and “Stress response/Protein folding/Ubiquitination” (B13). In agreement with the DEG analysis in Fig. 2, there was a clear upregulation in gene expression in those modules indicating myeloid responses or the consequences of myeloid activation and interferon signalling; “Macrophages/Cytokine signalling” (B5), “Myeloid cells/Lipid metabolism” (B6), “Myeloid cells/Granulocytes” (B7, B17, B18), “Ifn-signalling/Il10” (B11), and “Ifn-signalling” (B14). In addition, there was a module associated with “T cell differentiation” (B37) that was downregulated in the RMT infections as compared to SBP.

Investigation of the top 20 genes contributing to the enriched modules revealed a number of genes similar to those found in the pairwise comparisons such as *ifng*, *ccl2/mcp1, icam1, nos2, cxcl9/mig, cxcl10/ip10,* and *gbps*, as well as a number of additional genes like eg. *lys2, csf1*, and *mmps* (Fig. 4B). A number of additional cytokines and chemokines or their receptors (*il6, il15ra, ccl4/mip1b, cxcl1/kc/groa, and cxcl2/mip2)* were identified in the modules related to the immune system. In summary, this modular analysis indicates an increase in activation of myeloid cells and interferon signalling in RMT infections compared with SBP infections on day 6 post-infection.

In addition to pairwise comparisons of differential gene expression we looked for changes in kinetics within the time series, using a Likelihood Ratio Test (LRT) to compare the RMT and SBP infections for these changes. Here we identified a number of genes that followed a unique pattern in RMT infections on day 6 of infection (Supplementary Fig. S6). These results showed a similar set of genes associated with mainly myeloid cells (*lyz2, nos2, nfkb2, cd86, nfkbia, csf1, csf2rb, tlr2, neu1*), and genes encoding chemokines and cytokines (*il1rn, ccl2/mcp1*), and few genes downstream of interferon signalling (*irf8, icam1, irf2bp2*) as both the pairwise comparison and modular analysis, reinforcing the transcriptional signature identified in both analyses.

### 3.4 Attenuated *P. chabaudi* infection triggers stronger cytokine and chemokine production in the spleen

**Fig. 5:**
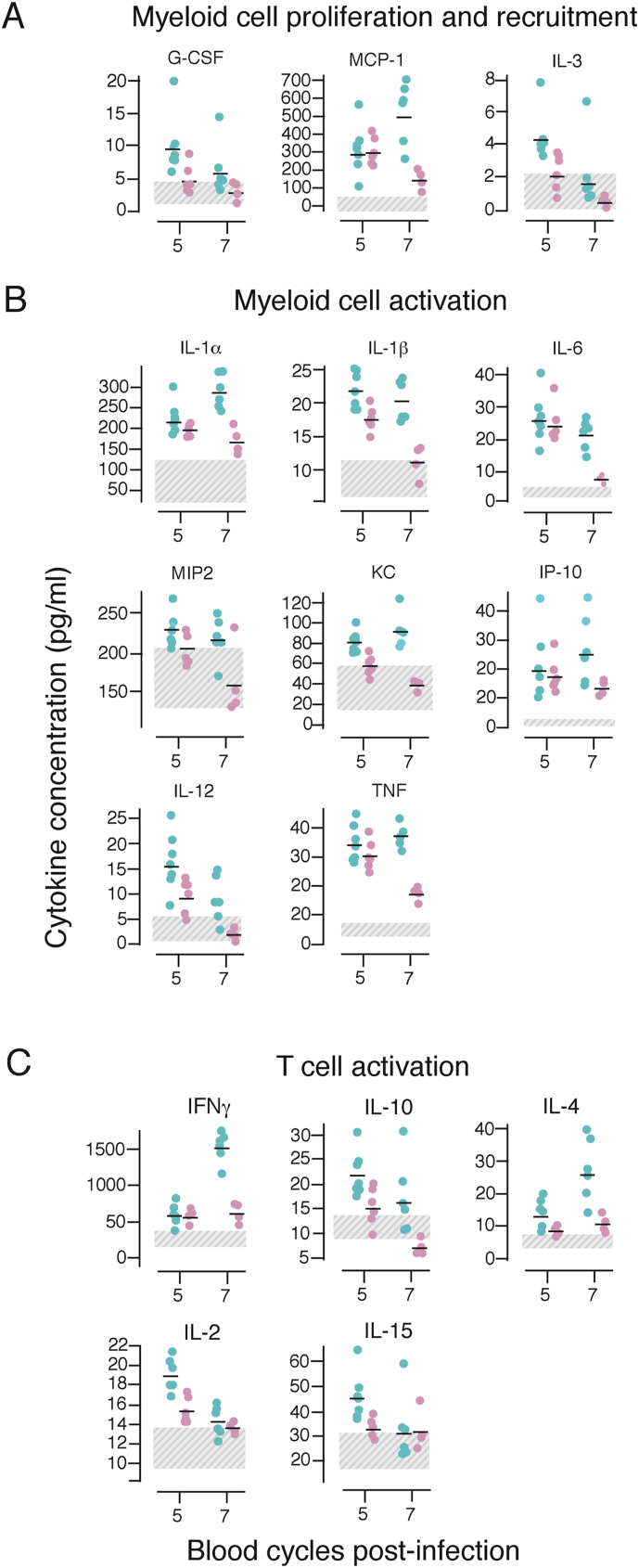
RMT *P. chabaudi* blood-stage infections trigger a stronger inflammatory response in the spleen. Spleen lysates were prepared from C57Bl/6J mice infected with 10^5^ RMT (blue symbols) or SBP-iRBCs (pink symbols) or from naïve controls at different days post-infection (shown are day 5 and 7) and investigated with a cytokine/chemokine protein array. Graphs depict protein content expressed as pg/ml for cytokines involved in myeloid cell proliferation and recruitment (**A**), myeloid cell activation (**B**) and T cell activation **(C)**. Graphs illustrate data from one experiment with 6 mice/group. The dashed areas denote the protein content of uninfected control samples. Transmission groups were compared per day via a nested ANOVA (see materials and methods section and Supplementary Table 5). All comparisons shown have a *p*-value below 0.05.

Both differential gene expression and modular analyses revealed that the acute phase of an attenuated RMT *P. chabaudi* blood-stage infection resulted in greater upregulation of genes encoding cytokines and chemokines as well as genes involved in activation and responses of myeloid cells, when compared with an SBP *P. chabaudi* infection. To determine whether any of the chemokines or cytokines observed at the RNA level were also produced as proteins, we measured 27 chemokines and cytokines in lysates of whole spleens from mice infected with RMT or SBP *P. chabaudi*-iRBCs at several timepoints (days 1–7 of infection) using a multiplex cytokine array. No cytokines/chemokines could be detected in spleen lysates above the level of naïve controls on days 1–3 of either blood-stage infection (data not shown). However, 16 were present in higher amounts in spleen lysates from the RMT infection compared with lysates from SBP-infected spleens on days 5 and/or 7 of infection (Fig. 5, Supplementary Fig. S7 and Supplementary Table 5). Most of those can be produced by macrophages and monocytes and many of them are dependent on IFNγ and NF-κB signalling, including IL-1, IL-6, IL-12, TNF, CXCL1/KC/GROα, CXCL10/IP-10, and CCL2/MCP-1. T cell cytokines IL-2, IL-15, IFNγ, IL-10, IL-4, and IL-9 were also present in greater amounts in RMT spleens. Four cytokines/chemokines were present at similarly increased levels in lysates from both infections (CXCL9/MIG, CCL5/RANTES, LIF, and VEGF), and IL-7 similarly decreased in both infections (Supplementary Fig. S7). Only two chemokines, CCL3/MIP-1α and CCL4/MIP-1β were higher in lysates from SBP infections compared with RMT on day 7 (Supplementary Fig. S7).

Together these data support the RNAseq results and suggest that RMT-iRBCs trigger a stronger myeloid and IFNγ-dependent inflammatory response in the spleen that may be important in attenuating *P. chabaudi* RMT infections.

### 3.5 Resident macrophages, but not recruited inflammatory monocytes, contribute to the control of attenuated mosquito-transmitted *P. chabaudi* infections

The RNAseq data together with the analysis of chemokines and cytokines produced in the spleen demonstrate a very strong link between early myeloid cell responses in RMT blood-stage infections and attenuation of parasitaemia.

To determine whether there is a role for myeloid cells in attenuating RMT *P. chabaudi* infections, mice were treated with clodronate liposomes two days prior to infection (D-2) and subsequently at three-day intervals to deplete myeloid cells (Brugat et al., 2017; van Rooijen and van Nieuwmegen, 1984). Two days after the first dose, i.e. at the time of infection with RMT or SBP-iRBCs (D0), flow cytometric analysis demonstrated that red pulp macrophages, CD169^+^ marginal metallophilic macrophages, and CCR2^+^ Ly6C^+^ inflammatory monocytes were fully depleted, and MARCO^+^ marginal zone macrophages and CCR2^-^ Ly6C^+^ monocytes partially depleted. There was little effect on neutrophils, eosinophils and dendritic cells after one injection (Supplementary Fig. S8). Analysis of the splenic myeloid populations at day 10 of infection (D10), three days after the fourth injection of clodronate liposomes, revealed that, in addition to red pulp macrophages, marginal metallophilic macrophages and CCR2^+^ inflammatory monocytes, dendritic cells were also fully depleted, and neutrophils, eosinophils, marginal zone macrophages and CCR2^-^ monocytes partially depleted.

**Fig. 6:**
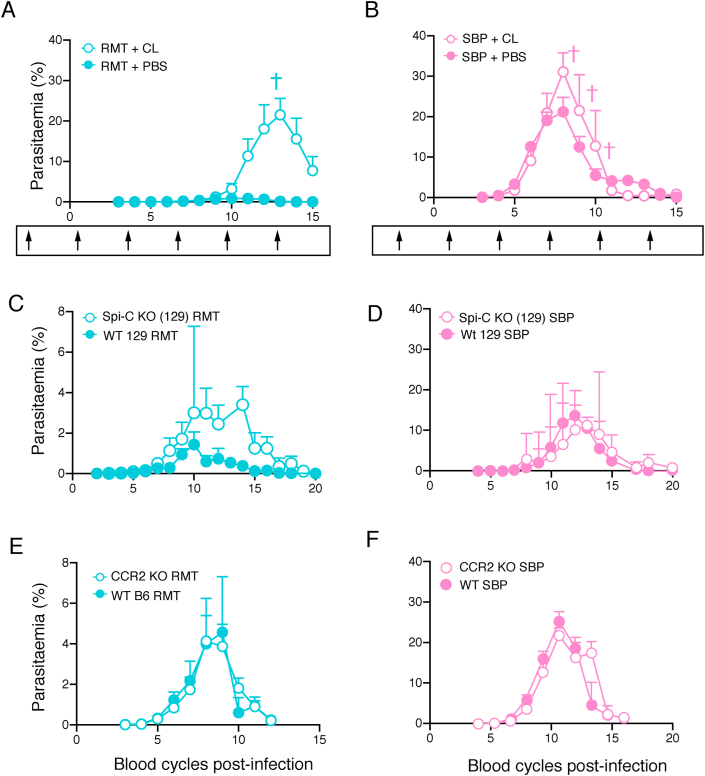
Myeloid cells are important for protection against attenuated *P. chabaudi*. **A-B.** Course of a *P. chabaudi* blood-stage infection in C57Bl/6J mice treated with 200 uL clodronate liposomes (CL, open symbols) i.v. or control liposomes (PBS, closed symbols) and infected by injection of 10^5^ RMT-iRBCs (**A**) or SBP-iRBCs (**B**). Arrows underneath each graph illustrate time points of liposome injection (first injection on day −2 and after that every three days). **C–F.** Course of a *P. chabaudi* blood-stage infection in Spi-C knockout mice on a 129 background (open symbols) and in wild-type 129 S/J and 129SvEv mice (WT 129, closed symbols) **(C**–**D),** or in CCR2 knockout mice (open symbols) and wild-type C57Bl/6J (WT B6) mice (closed symbols) **(E**–**F)** infected via injection of 10^5^ RMT-iRBCs **(C, E)** or SBP-iRBCs **(D, F)**. The graphs show mean (+/- SEM) of percentage parasitaemia calculated from log-transformed data. Shown are data from one representative experiment with 5–7 mice/group **(A**–**B)**, n = 11 mice (pooled data from six individual experiments; **C)**, data from one experiment with 3–4 mice/group **(D)**, and n = 7–12 mice (pooled data from two experiments; **E-F)**.

This treatment regimen resulted in a very marked exacerbation of the acute RMT *P. chabaudi* infection; peak parasitaemia of approximately 30 % in clodronate-treated RMT-infected mice, compared with a parasitaemia less than 5 % in untreated mice or mice treated with empty liposomes (*p* < 0.0001; Fig. 6A). Clodronate treatment of mice infected via mosquito bite (MT) showed a similar exacerbation of the peak parasitaemia (*p* < 0.0001; Supplementary Fig. S9A). By contrast, clodronate treatment had a much smaller effect on SBP infections in the same time period of infection (*p* = 0.0453; Fig. 6B). Similar exacerbation of RMT infections in C57Bl/6J mice were observed when mice were given only 2 doses of clodronate at day −2 and day 7 (data not shown). Together these data suggest that myeloid cells are much more important in controlling acute attenuated *P. chabaudi* blood-stage infections, than in controlling SBP infections.

As red pulp macrophages and CCR2^+^ inflammatory monocytes were both fully depleted by clodronate treatment, we examined whether either of these cell populations were involved in the attenuation of acute infection. For this we made use of Spi-C and CCR2 knockout mice. The Spi-C transcription factor is important for the development of red pulp macrophages (Kohyama et al., 2009) and mice deficient for Spi-C do not have red pulp macrophages in the spleen [(Kohyama et al., 2009) and Supplementary Fig. S9B]. In Spi-C knockout mice, lack of red pulp macrophages resulted in a slightly increased peak (*p* = 0.0037) and severely delayed parasite clearance (Fig. 6C), indicating that red pulp macrophages contribute to protection against RMT parasites. Our data contrast with the previously described lack of effect in SBP *P. chabaudi* infections (Kim et al., 2012), and shown here (*p* = 0.5972; Fig. 6D). Total spleen cell numbers were similar in infected Spi-C and wild-type 129SvEv mice (Supplementary Fig. S9C), but with increased numbers of CD169^+^ marginal metallophilic macrophages, MARCO^+^ marginal zone macrophages and CCR2^+^ inflammatory monocytes, suggesting that other myeloid cells could compensate for the lack of red pulp macrophages in Spi-C knockout mice.

We previously described the influx of inflammatory monocytes from the bone marrow into the spleen in a CCR2-dependent manner in SBP *P. chabaudi* infections (Sponaas et al., 2009). This influx is also observed in RMT infections (Supplementary Fig. S8C). However, despite the fact that no CCR2^+^ inflammatory monocytes were detected in the spleen of CCR2 knockout mice (Supplementary Fig. S9D), these mice had similar peak parasitaemias as their wild-type C57Bl/6J mice for both infections initiated with RMT and SBP *P. chabaudi* [*p* = 0.9486 and *p* = 0.2990 respectively; Fig. 6E–F and (Sponaas et al., 2009)], indicating that bone marrow-derived CCR2^+^ inflammatory monocytes are not involved in attenuation of infection. As CCR2^-^ monocytes were only slightly reduced upon clodronate treatment, these results together with those from the CCR2 knockout mice indicate that monocytes do not contribute to protection.

Together, these results indicate that tissue-resident macrophages, including red pulp macrophages, but not recruited inflammatory monocytes, are important for the attenuation of RMT *P. chabaudi* infections.

### 3.6 Disruption of IFNγ-signalling increases acute parasitaemia in mice infected with RMT *P. chabaudi*

In addition to the strong myeloid signature found in RMT *P. chabaudi* infections compared with SBP infections, there were also two modules that suggested earlier IFN responses in RMT infections (Fig. 4), and higher amounts of IFNγ and IFNγ-related molecules were produced by the spleens during RMT infection both at the RNA (Fig. 3, Fig. 4 and Supplementary Fig. S6) and protein (Fig. 5 and Supplementary Fig. S7) level. In both RMT and SBP *P. chabaudi* infections shown here, many lymphoid cell populations produce IFNγ in the first 7 days of infections (Supplementary Fig. S10A-B), with CD4^+^ T cells comprising the greatest numbers and proportions by day 7 of infection. Small differences were observed between groups in that spleens from SBP-infected mice had more T cells producing IFNγ on day 5 (CD4^+^ T cells) or on day 7 (CD8^+^ T cells), whereas there were fewer γδ NK1.1^+^ NKT cells producing IFNγ on day 5 compared to mice infected with RMT parasites. As IFNγ signalling is important for macrophage activation and plays a role in eliminating blood-stage *Plasmodium* infections in mice (Brugat et al., 2014; Kim et al., 2012; Meding et al., 1990; Stevenson et al., 1990; Su and Stevenson, 2000; van der Heyde et al., 1997), we investigated whether IFNγ-signalling was also important for the attenuation of the acute RMT *P. chabaudi* infection.

**Fig. 7:**
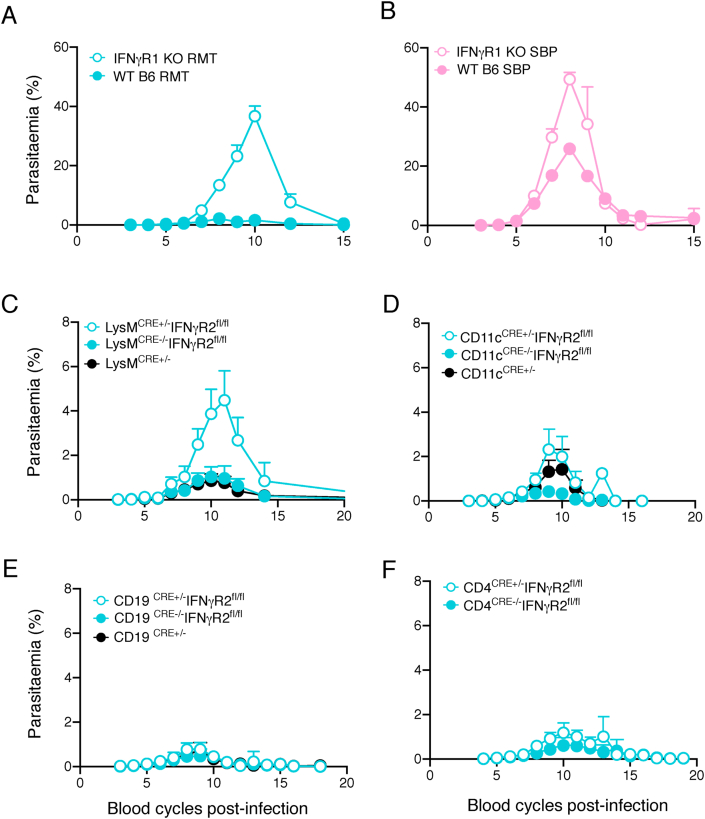
IFNγ-signalling, more specifically in myeloid cells, contributes to the anti-parasite response with RMT parasites. Course of a *P. chabaudi* RMT blood-stage infection in IFNγR1 knockout (KO) mice **(A**–**B)** or in conditional LysM^Cre^IFNγR2^fl/fl^**(C)**, CD11c^Cre^IFNγR2^fl/fl^**(D)**, CD4^Cre^IFNγR2^fl/fl^**(E)**, or CD19^Cre^IFNγR2^fl/fl^**(F)** knockout mice infected with 10^5^ RMT-iRBCs **(A, C–F)** or SBP-iRBCs **(B)**. Each graph in panel **C–F** compares heterozygous experimental mice (Cre+/-, open symbols) with their wildtype littermates (Cre−/−, closed symbols) or heterozygous Cre mice without the IFNγR2^fl/fl^ (black). The graphs show mean (+/- SEM) of percentage parasitaemia calculated from log-transformed data. Shown are pooled data from two individual experiments (n = 8–10 mice; **A-B),** four experiments (n = 14–17 mice; **C)**, three experiments (n = 4–17 mice; **D)**, four experiments (n = 7–17 mice; **E)** or from four experiments (n = 20–21 mice; **F)**.

Mice lacking the IFNγ receptor (IFNγR), through a genetic deletion of the *ifnγr1* gene, the IFNγ-binding subunit, were infected with either RMT or SBP *P. chabaudi*-iRBCs. In the absence of IFNγR-signalling there was a transiently increased acute parasitaemia in both SBP and RMT infections (Fig. 7A–B), and when infections were initiated via mosquito bite (*p* < 0.0001; Supplementary Fig. S10C). However, the increase in RMT parasitaemia in the knockout was very striking resulting in an average peak of approximately 40 % (an increase of approximately 8-fold; *p* = 0.0001). In the SBP infection, peak parasitaemia increased only approximately 2-fold in the absence of IFNγR signalling (*p* < 0.0001) as described previously (Brugat et al., 2014; Kim et al., 2012; Meding et al., 1990; Stevenson et al., 1990; Su and Stevenson, 2000; van der Heyde et al., 1997). From these data, we conclude that signalling through the IFNγ receptor contributes to the attenuation of infection observed in *P. chabaudi* RMT infections.

We next made use of mice in which the IFNγR2 chain, essential for transducing the IFNγ signal (Hemmi et al., 1994; Lee et al., 2015), was selectively deleted on two different populations of myeloid cells: *LysM*^*cre*^^*+/-*^ *Ifnγr2*^*fl/fl*^ (granulocytes, monocytes and macrophages), and *CD11c*^*cre+/-*^ *Ifnγr2*^*fl/fl*^ (classical and plasmacytoid dendritic cells, some B cells); and compared the infections with those in mice in which IFNγ-signalling in CD4^+^ and CD8^+^ T cells (*CD4*^*cre+/-*^ *Ifngr2*^*fl/fl*^) or B cells (*CD19*^*cre+/-*^*Ifnγr2*^*fl/fl*^) was deleted. The acute *P. chabaudi* parasitaemias in *LysM*^*cre ±*^^*+/-*^ *Ifnγr2*^*fl/fl*^ mice had significantly higher peak parasitaemias than their *LysM*^*cre−/−*^ *Ifnγr2*^*fl/fl*^ (*p* = 0.0105) and *LysM*^*cre+-/-*^ (*p* =0.0005) littermate control mice (Fig. 7C). Peak parasitaemias were similar in *CD11c*^*cre*+/-^ *Ifnγr2*^fl/fl^ mice, *CD4*^*cre+/-*^ *Ifnγr2*^*fl/fl*^ mice or *CD19*^*cre+/-*^ *Ifnγr2*^*fl/fl*^ mice compared with *their respective Cre^+/-^* *or fl/fl* control mice (Fig. 7D–G). These data indicate that IFNγ-signalling in granulocytes, macrophages and monocytes may contribute to the anti-parasitic response in RMT infections. These cell types are also found to be depleted with the clodronate liposome injections (see above).

## 4 Discussion

Mouse models of malaria are routinely used to understand the immune response induced by the *Plasmodium* parasite. Human infections occur via mosquito bite, whereas most laboratory infections that investigate immune responses to the blood-stage of infection generally use injected SBP-iRBCs to initiate infections. We have previously shown that infections with the rodent malaria parasite, *Plasmodium chabaudi chabaudi* AS in mice, initiated via infected mosquito bites (MT), or with blood-stage parasites recently derived from a mosquito-transmitted infection (RMT) have lower blood-stage parasitaemias and less pathology compared with infections caused by SBP *P. chabaudi-*iRBCs. As shown here and previously (Brugat et al., 2017; Spence et al., 2013), this reduction of parasitaemia is brought about by interactions with the immune system of the mammalian host. If we are to use mouse models to infer human immune responses in malaria, then it is important to understand the host response induced by parasites that more closely resemble those of naturally-transmitted infections, and to find out how the responses differ from those induced by the more commonly used SBP parasites.

Here we show that the attenuated infection course seen with MT *P. chabaudi* was not the result of responses to the pre-erythrocytic stages of the parasite but to the blood-stages themselves. Blood-stage parasites derived from an MT infection induced stronger myeloid and IFN responses in the spleen than SBP parasites, and these responses were important for the control of acute blood-stage infection. Depletion of myeloid cell populations and more specifically red pulp macrophages, lack of IFNγ-signalling, and deletion of the IFNγ receptor on myeloid cells all resulted in more severe blood-stage parasitaemias that more closely resemble SBP *P. chabaudi* infections.

Before a blood-stage infection is established, MT *Plasmodium* infections expose the mammalian host to products of mosquito salivary glands, as well as the pre-erythrocytic stages of the parasite, which all induce immune responses (Barros et al., 2016; Belnoue et al., 2008; Bizzarro et al., 2013; Depinay et al., 2006; Donovan et al., 2007; Fonseca et al., 2007; Kurup et al., 2019; Liehl et al., 2015; Machain-Williams et al., 2012; Ribot et al., 2019). These responses could influence the subsequent course of the blood-stage infection, resulting in the attenuation observed in MT *P. chabaudi* infections. However, in our experiments we showed that the contribution of these responses to reduce blood-stage parasitaemia can be ruled out, as infections initiated by *P. chabaudi*-iRBCs derived from an MT infection (RMT), obviating the bite and pre-erythrocytic stages, also gave rise to an attenuated infection. We have shown previously that at least four direct blood passages of *P. chabaudi* are required before the typical course of infection of an SBP infection is observed (Brugat et al., 2017; Spence et al., 2013), suggesting that MT or RMT *P. chabaudi* iRBCs induce host immune responses that substantially reduce acute parasitaemias.

Interactions between *Plasmodium-*iRBCs and the host immune system are most likely to take place in the spleen [reviewed in (Del Portillo et al., 2012; Engwerda et al., 2005)]. This has been shown to be an important lymphoid organ for the development of immunity to *Plasmodium* in rodent malaria models, as shown here in MT *P. chabaudi* infections, and by others in SBP infections (Chotivanich et al., 2002; Del Portillo et al., 2012; Engwerda et al., 2005; Kumar et al., 1989; Yap and Stevenson, 1994). The spleen however is not the only organ involved in protective immunity as splenectomised mice still are able to control infection albeit at a higher parasitaemia levels, unlike Rag1 knockout mice, indicating that other lymphoid organs such as lymph nodes and liver also play a role. Comparative transcriptomics of spleen samples taken from early RMT and SBP blood-stage infections by pairwise comparison, modular analysis and Likelihood Ratio Testing revealed stronger and earlier myeloid and IFN signatures in RMT infections in the acute blood-stage infection. Differential gene expression analyses also identified genes encoding cytokines, cytokine receptors, chemokines and interferon-inducible factors, which were more strongly induced in RMT infections. In line with these observations, multiple cytokines and chemokines such as IL-1α, IL-1β, IL-6, IL-10, IFNγ, MCP-1, KC, IP-10 and others were present at higher level in lysates prepared from spleens taken from mice undergoing an RMT infection compared with those with an SBP infection. Together, these results showed that RMT *P. chabaudi* triggered an overall stronger and earlier innate and IFNγ-dependent immune response in the spleen, which could be responsible for the attenuation of the blood-stage infection.

The strong myeloid signature associated with RMT infections seen in the transcriptomic analysis suggests that myeloid cells may play a key role in the early control of these parasites and thus attenuation. Phagocytic cells, such as monocytes and macrophages, have long been known to be essential as a first line of innate defence in immunity against blood-stage malaria both as producers of cytokines and chemokines and as effector cells [reviewed in (Deroost et al., 2016)]. Infected RBCs are phagocytosed by myeloid cells by both antibody-dependent and-independent mechanisms in *Plasmodium* infections of mice and humans. There are many different myeloid populations in the spleen often with overlapping cell surface markers and functions (Borges da Silva et al., 2015; Engwerda et al., 2005), and the question is whether any particular cell population(s), tissue-resident or migrating, is more important in reducing parasitaemias early in the course of MT/RMT *P. chabaudi* blood-stage infections. We found that multiple injections of clodronate into mice to remove myeloid cells (van Rooijen et al., 1989; van Rooijen and van Nieuwmegen, 1984) led to an approximately 10-fold increase in peak parasitaemia of RMT infections, in contrast to the much smaller effects seen in SBP *P. chabaudi* infections. Clodronate treatment before and during infection selectively removed both bone marrow-derived CCR2^+^ inflammatory monocytes and tissue macrophages such as red pulp macrophages and CD169^+^ marginal metallophilic macrophages, and also partially depleted MARCO^+^ marginal zone macrophages, eosinophils and neutrophils (Ferenbach et al., 2012; Lundmark et al., 2013; van Rooijen et al., 1989; van Rooijen and van Nieuwmegen, 1984). Although there was a clear CCR2-dependent influx of bone-marrow-derived inflammatory monocytes in RMT *P. chabaudi* infections similar to that observed previously in SBP infections (Sponaas et al., 2009), which may have iRBC phagocytic activity, we ruled out a role for these cells as lack of CCR2 in knockout mice had no impact on the magnitude or duration of an acute RMT infection.

Our results suggest that tissue macrophages in the spleen contribute to attenuation of RMT-infection. Red pulp macrophages, along with marginal zone and marginal metallophilic macrophages comprise the majority of the splenic tissue macrophage population, and in these experiments were very effectively depleted by clodronate treatment with the exception of marginal zone macrophages, which were only partially depleted. Red pulp macrophages are localised in the red pulp where *Plasmodium* parasites can also be found (Yadava et al., 1996) and are involved in the removal of senescent RBCs and are therefore the most likely candidates. To investigate the role of red pulp macrophages, we used Spi-C knockout mice. These mice lack red pulp macrophages because they lack the transcription factor Spi-C that selectively induces formation of red pulp macrophages (Kohyama et al., 2009). Our study demonstrates that Spi-C knockout mice infected with RMT parasites, but not SBP parasites, had increased peak parasitaemias and significantly prolonged acute parasitaemias, which eventually resolved. These data support the idea that red pulp macrophages are contributing to the control of *Plasmodium* parasites in the attenuated RMT infection. By contrast, and in agreement with a previous study by Kim et al. (2012), the lack of red pulp macrophages had little effect on the outcome of an SBP infection. It appears then that RMT and SBP parasites either differentially activate red pulp macrophages, and/or are differentially susceptible to their effects. Splenic red pulp macrophages are reduced in numbers at day five of the *P. chabaudi* blood-stage infection in agreement with Nahrendorf et al. (2021). This is not incompatible with a role for these cells in early control of RMT parasitaemias, as attenuation of the infection is already taking place at this time. The subsequent loss of these cells, or their loss of classical surface markers may be a result of their activation and/or death after parasite killing. They are subsequently replenished as shown by Nahrendorf et al. (2021).

As the increased parasitaemia brought about by the lack of red pulp macrophages was much less than that seen after clodronate depletion, other macrophages are obviously required. There are very few reports for a protective role of other tissue-resident macrophages in rodent malarias. One study demonstrated a role for CD169^+^ macrophages in limiting *P. berghei* blood-stage pathology and sequestration, allowing mice to recover from an otherwise lethal infection (Gupta et al., 2016). These macrophages were also depleted by the clodronate treatment in our study, although, as they are situated at the inner border of the marginal zone, they are less likely to interact with *P. chabaudi*-iRBCs that are mainly found in the red pulp. MARCO^+^ marginal zone macrophages were only partially depleted by our treatment, but they are situated at the marginal zone lining the red pulp. We found that these cells were also increased in Spi-C knockout mice, and, therefore, could potentially contribute to the elimination of iRBCs.

IFNγ enhances macrophage activation, inducing proinflammatory cytokines, thus promoting the inflammatory innate response and subsequent adaptive immune response, and at the same time enhancing macrophage phagocytic ability and cytolytic potential to eliminate pathogens [reviewed in (Deroost et al., 2016; Mosser and Edwards, 2008; Murray and Wynn, 2011)]. Induction of interferons, interferon-regulatory factors (IRFs) and interferon-stimulated genes (ISGs) has long been associated with blood-stage malaria infections. *Plasmodium falciparum* in humans induces upregulation of IRF1, 5, 7, 8 and 9, and *P. vivax* is associated with increased expression of IRF1 and 7 in peripheral blood mononuclear cells, as well as increased expression of Type I and II interferons (Gun et al., 2014; Mangano et al., 2008; Vallejo et al., 2018). In the case of *P. falciparum* infections, *irf1* expression has been associated with control of the parasite (Mangano et al., 2008). In *Plasmodium* infections of mice initiated with SBP parasites, IFN-dependent and macrophage responses in the spleen are important in controlling acute blood-stage parasitaemia, with IFN signatures, ISG sand type I IFNs upregulated (Araujo et al., 2012; Favre et al., 1997; Gun et al., 2014; Kim et al., 2008, 2012; Li et al., 1999; Meding et al., 1990; Su and Stevenson, 2000; Yoneto et al., 1999). Although the numbers of splenic IFNγ-producing NK, NK1.1^+^ NKT, γδ T cells and αβ T cells observed here in RMT and SBP *P. chabaudi* infections within the first 7 days of infection were similar, our comparative transcriptome analysis showed small but consistently higher expression levels of a number of *irf* genes, such as *irf1,* which mediates signalling of type I/II IFNs, and regulates antigen-presentation, and *irf7* which is a master regulator of Type I IFN (Negishi et al., 2018). Furthermore, using a modular analysis of gene expression (Singhania et al., 2019), RMT infections induced IFN and macrophage modules earlier than SBP infections, as well as greater amounts of inflammatory cytokines and chemokines in spleen lysates.

It was previously observed in SBP *P. chabaudi* infections, that lack of IFNγ-signalling led to exacerbated acute *P. chabaudi* parasitaemias (Brugat et al., 2014; Kim et al., 2012; Meding et al., 1990; Stevenson et al., 1990; Su and Stevenson, 2000; van der Heyde et al., 1997). Here, deletion of the IFNγR1 subunit had a substantially more profound impact on the acute infection of RMT/MT *P. chabaudi* infections, than was observed in SBP infections, showing that an early IFNγ response is important in attenuating parasitaemia. Control of RMT infections was also compromised in conditional knockout mice, in which the IFNγR2 subunit was deleted on LysM-expressing myeloid cells, but not CD11c^+^ cells. LysM is expressed in monocytes, macrophages, granulocytes and some dendritic cells (Abram et al., 2014). In the spleen, 60–80 % of neutrophils are LysM^+^ in contrast to only 40 % of spleen red pulp or marginal zone macrophages. The increase in parasitaemia in the LysM^c^^+/-^ IFNγR2^fl/fl^ mice was not as strong as that seen after clodronate treatment. This may be because the penetrance of the LysM *cre* drivers was not complete in these cells (Shi et al., 2018), or that other myeloid cells, not expressing LysM, contributed to parasite control. None of the Cre drivers targeting macrophages discriminate clearly between individual populations of macrophages, depletion efficiency and targeting specificity for endogenous macrophages due to the lack of specific markers or transcriptional factors and the large heterogeneity of the population (Shi et al., 2018). Further detailed study on the contribution of other resident macrophages when such tools become available would be required to determine which cells receiving an IFNγ signal could be important in parasite clearance; however, our study revealed that an IFN signal probably through tissue-resident macrophages including red pulp macrophages was required for attenuation of RMT infections.

Our study suggests that myeloid cells and IFNγ responses were activated earlier or more strongly by blood-stage parasites that have recently been transmitted through mosquitoes. The faster timing and/or magnitude of this response is likely to lead to clearance of parasites more effectively, preventing development of higher parasitaemias. How do RMT parasites stimulate this earlier response? MT/RMT and SBP blood-stage parasites are transcriptionally different (Spence et al., 2013), suggesting that there may be components of RMT/MT parasites that are more effective in activating the innate immune system and IFNγ. The major differentially transcribed genes between MT/RMT and SBP *P. chabaudi* are members of one multigene family - the *pir* multigene family (Brugat et al., 2017; Spence et al., 2013). The function(s) of these genes is unknown, although it has been speculated that they are involved in immune evasion and sequestration [reviewed in (Cunningham et al., 2010)]. SBP *P. chabaudi*-iRBCs have a very reduced repertoire of *pir* genes transcribed, and lower *pir* gene expression overall, when compared with MT/RMT parasites early in acute infections. By contrast, MT/RMT parasites express a wider repertoire of *pirs* and they are expressed at higher levels. It is tempting to speculate that some of those RMT/MT-associated *pirs* are responsible for inducing a splenic host response that attenuates infection. Uncovering a mechanism for this would provide important information about host resistance and virulence in blood-stage *Plasmodium* infections initiated by naturally-transmitted parasites.

## CRediT authorship contribution statement

**Katrien Deroost:** Writing – original draft, designed and performed the experiments with experimental help from Caroline Hosking, wrote the manuscript. **Christopher Alder:** Formal analysis, Writing – original draft, performed the bioinformatic analyses, wrote the manuscript. **Caroline Hosking:** carried out the splenectomies. **Sarah McLaughlin:** carried out the splenectomies. **Jing-Wen Lin:** Formal analysis, performed the bioinformatic analyses. **Matthew D. Lewis:** helped with the clodronate experiments. **Yolanda Saavedra-Torres:** carried out the splenectomies. **John W.G. Addy:** Formal analysis, performed the statistical analyses of the splenic cytokines. **Prisca Levy:** helped with the mosquito transmission of Plasmodium chabaudi. **Maria Giorgalli:** Formal analysis, performed the bioinformatic analyses. **Jean Langhorne:** Writing – original draft, wrote the manuscript.

## Declaration of competing interest

The authors declare that they have no known competing financial interests or personal relationships that could have appeared to influence the work reported in this paper.

## Appendix A Supplementary data

Multimedia Component 1 **Supplementary Figure S1. Dendogram of sample correlation.** Individual samples were organised into a dendogram to see how well the different samples correlated within a group. The red squares indicate the two samples that were removed from the analysis (RMT.R1 from D4 and SBP.R2 from D4) as they were found not to be infected. D, day of the blood-stage infection; R, replicate. Multimedia Component 1

Multimedia Component 2 **Supplementary Figure S2. The difference in parasitaemia is due to the immune response but B cells are not involved.** Course of a *P. chabaudi* blood-stage infection in Rag1 knockout mice after intraperitoneal (i.p.) injection of 10^5^ RMT-iRBCs (blue) or 10^5^ SBP-iRBCs (pink) (**A**), μMT (open symbols) and wild-type C57Bl/6J mice (closed symbols) after intravenous (i.v.) injection of 100 sporozoites (SPZs) (**B**) or 10^5^ SBP-iRBCs i.p. (**C**). The graphs show mean (+/- SEM) of percentage parasitaemia calculated from log-transformed data. Shown are data from one individual experiment with 8 mice/group **(A)** or 7 mice/group **(B-C)**. Multimedia Component 2

Multimedia Component 3 **Supplementary Figure S3. No differences in the development of splenomegaly between RMT and SBP spleens in *P. chabaudi* blood-stage infections.** Differences in splenomegaly between infection routes were investigated in three different ways: by comparing spleen weights (**A**), spleen sizes (length*width) (**B**), and splenocyte numbers (**C**) at different time points during the early infection phase from 7-10 weeks old C57Bl/6J mice infected by mosquito bite (MT) (green), i.v. injection of 100 SPZs (black), i.p. injection of 10^5^ RMT-iRBCs (blue) or i.p. injection of 10^5^ SBP-iRBCs (pink). Data were normalised by square root or log-transformation and spleen weight and size were normalised to the total body weight (BW) of the respective mouse from which the spleen was removed. Shown are pooled data from two experiments with 5-6 mice/group (MT), three to six experiments with 3-7 mice/group (RMT and SBP), or from one-three experiments with 5-6 mice/group (SPZ). Two-way ANOVA with Tukey's multiple comparisons test has been performed to test for differences between groups on a specific day. Lines above groups with asterisks illustrate significances between groups. *, *p* < 0.05; **, *p* < 0.01; ***, *p* < 0.001; ****, *p* < 0.0001. Multimedia Component 3

Multimedia Component 4 **Supplementary Figure S4. No differences in total T and NK cell numbers in the spleen.** C57Bl/6J mice were infected with 10^5^ RMT-iRBCs or SBP-iRBCs or left uninfected. At the indicated time points after infection, mice were sacrificed, and their spleens removed and homogenised to obtain a single cell suspension. Cells were labelled with specific fluorescently-labelled antibodies as described in the materials and methods section and investigated by flow cytometry. **A.** Gating strategy for the investigated cell types. **B.** Total cell numbers of the indicated cell types in RMT (blue symbols), SBP (pink symbols) or uninfected control (grey dashed areas) spleens. Graph shows data from one individual experiment with 6 mice/group. Unpaired Mann-Whitney U tests were calculated to look for differences between RMT and SBP groups. Lines above groups with asterisks illustrate significances between groups. *, *p* < 0.05. NK(T) cells, natural killer (T) cells. Multimedia Component 4

Multimedia Component 5 **Supplementary Figure S5. No difference in splenic myeloid cells numbers.** C57Bl/6J mice were infected with 10^5^ RMT-iRBCs or SBP-iRBCs or left uninfected. At the indicated time points after infection, mice were sacrificed, and their spleens removed and homogenised to obtain a single cell suspension. Cells were labelled with specific fluorescently-labelled antibodies as described in the materials and methods section and investigated by flow cytometry. **A.** Gating strategy for the investigated cell types. **B.** Total cell numbers of the indicated cell types in RMT (bue symbols), SBP (pink symbols) or uninfected control (grey dashed areas) spleens. Graph shows data from one experiment with 6-7 mice/group. Unpaired Mann-Whitney U tests were calculated to look for differences between RMT and SBP groups but none of the comparisons were significant. RpMFs, red pulp macrophages; MZMs, marginal zone macrophages; MMMs, marginal metallophilic macrophages; iMos, inflammatory monocytes; Mos, monocytes; DCs, dendritic cells. Multimedia Component 5

Multimedia Component 6 **Supplementary Figure S6. Heatmap of unique RMT genes identified via the Likelihood Ratio Test.** The Likelihood Ratio Test (LRT) was performed to look for changes in kinetics between RMT and SBP infections within the time series. The heatmap shows a number of genes that follow a unique pattern in RMT infections on day 6 of the infection in four uninfected control mice, four RMT infected mice and four SBP infected mice. Colours represent the Z-score values, which is a measurement of the number of standard deviations a sample value is above or below the mean across all samples for a given gene. Multimedia Component 6

Multimedia Component 7 **Supplementary Figure S7. Cytokines and chemokines in spleen lysates.** Spleen lysates were prepared from C57Bl/6J mice infected with 10^5^ RMT (blue symbols) or SBP-iRBCs (pink symbols) at different days post-infection (shown are day 5 and 7) or from naïve control mice (grey dashed areas) and investigated with a cytokine/chemokine protein array. Graphs depict protein content expressed as pg/ml for cytokines and chemokines that were not included in Figure 5. Graphs illustrate data from one experiment with 6 mice/group. The grey dashed areas denote the protein content of uninfected control samples. Transmission groups were compared per day via a nested ANOVA (see materials and methods section and Supplementary Table 5). Multimedia Component 7

Multimedia Component 8 **Supplementary Figure S8. Depletion of myeloid cells.** C57Bl/6J mice were treated with clodronate (CL) or control (PBS) liposomes starting 2 days before infection with 10^5^ RMT-iRBCs or SBP-iRBCs or left uninfected. Infected mice were treated with liposomes every 3 days. Mice were sacrificed at the day of infection (D0) after receiving one dose or 10 days post-infection (D10) after four doses of liposomes, and their spleens removed and homogenised to obtain a single cell suspension. Cells were labelled with specific fluorescently-labelled antibodies as described in the materials and methods section and investigated by flow cytometry. **Panel A-B** shows the gating strategy for the investigated cell types in control mice treated with one dose of control liposomes (PBS) **(A)** or clodronate liposomes (CL) **(B)** two days before. **Panel C** shows total cell numbers of the indicated cell types in RMT (blue bars), SBP (pink bars) or uninfected control (black bars) spleens. Graphs show data from one experiment with 4-5 mice/group (D0) and 3-8 mice/group for infected mice. Unpaired Mann-Whitney U tests were calculated to look for differences between PBS- and CL-treated groups. Asterisks denote significances compared to the PBS-treated group. *, *p* < 0.05; **, *p* < 0.01; ***, *p* < 0.001. Neu, neutrophils; DCs, dendritic cells; RpMFs, red pulp macrophages; MZMs, marginal zone macrophages; MMMs, marginal metallophilic macrophages; iMos, inflammatory monocytes; Mos, monocytes. Multimedia Component 8

Multimedia Component 9 **Supplementary Figure S9. Red pulp macrophages, but not recruited inflammatory monocytes, are important for attenuation of mosquito-transmitted *P. chabaudi* infections in C57Bl/6J mice. A.** Course of a *P. chabaudi* blood-stage infection in C57Bl/6J mice treated with 200 uL clodronate liposomes (CL) i.v. (open symbols) or untreated control mice (closed symbols) infected via mosquito bite. Arrows underneath the graph illustrate time points of CL injection (first injection on day -4, i.e. two days before the bite, and after that every three days). **B-D.** Mice were infected with 10^5^ RMT-iRBCs or left uninfected. At the indicated time points after infection, mice were sacrificed, and their spleens removed and homogenised to obtain a single cell suspension. Cells were labelled with specific fluorescently-labelled antibodies as described in the materials and methods section and investigated by flow cytometry. **Panel B and D** show the gating strategies for the investigated cell types in Spi-C knockout (KO) mice vs wild-type 129SvEv mice **(B)**, and in CCR2 knockout (KO) mice vs C57Bl/6J wild-type (B6 WT) mice **(D)**. Total cell numbers of the indicated cell types in RMT spleens on day 7 p.i. (D7, blue bars) or in uninfected control spleens (D0, black bars) are shown in **panel C**. The graph in panel A shows mean (+/- SEM) of percentage parasitaemia calculated from log-transformed data. Shown are data from one individual experiment with 9-13 mice/group. RpMFs, red pulp macrophages; MMMs, marginal metallophilic macrophages; MZMs, marginal zone macrophages; iMos, inflammatory monocytes; Mos, monocytes. Multimedia Component 9

Multimedia Component 10 **Supplementary Figure S10. No difference in the number of IFN**γ**-****producing cells in the spleen. A-B.** Mice were infected with 10^5^ RMT-iRBCs or SBP-iRBCs or left uninfected. At the indicated time points after infection, mice were sacrificed, and their spleens removed and homogenised to obtain a single cell suspension. Cells were labelled with specific fluorescently-labelled antibodies as described in the materials and methods section and investigated by flow cytometry. **(A)** Gating strategy for the investigated cell types. **(B)** Total cell numbers of the indicated cell types in RMT (blue symbols), SBP (pink symbols) or uninfected control (grey dashed areas) spleens. Graph shows data from one experiment with 6 mice/group. Unpaired Mann-Whitney U tests were calculated to look for differences between RMT and SBP groups. Lines above groups with asterisks illustrate significances between groups. **, *p* < 0.01; *, *p* < 0.05. **C.** Course of a *P. chabaudi* blood-stage infection initiated via mosquito-bite in IFNγR1 knockout (KO) mice (open symbols) compared to wild-type C57Bl/6J (WT B6) mice (closed symbols). Graphs show mean (+/- SEM) of percentage parasitaemia calculated from log-transformed data. Shown are data from one experiment with 13 mice/group. Multimedia Component 10

Multimedia Component 11 Supplementary Table 1: List of differentially expressed genes determined through pairwise comparison. Multimedia Component 11

Multimedia Component 12 Supplementary Table 2: 561 unique genes found to be differentially expressed in recently mosquito-transmittted (RMT) infected mice. Multimedia Component 12

Multimedia Component 13 Supplementary Table 3: 217 unique genes found to be differentially expressed in serially blood-passaged (SBP) infected mice. Multimedia Component 13

Multimedia Component 14 Supplementary Table 4: Gene content of the individual modules that are different between RMT and SBP. Multimedia Component 14

Multimedia Component 15 Supplementary Table 5: Statistical significances of cytokine/chemokine comparisons between groups. Multimedia Component 15
