# Supplementary figures and images for: Tissue macrophages and interferon-gamma signalling control blood-stage Plasmodium chabaudi infections derived from mosquito-transmitted parasites
Source: Curr Res Immunol. 2021 Jul 30;2:104–19. doi: 10.1016/j.crimmu.2021.07.002 (PMC8428512; doi:10.1016/j.crimmu.2021.07.002)

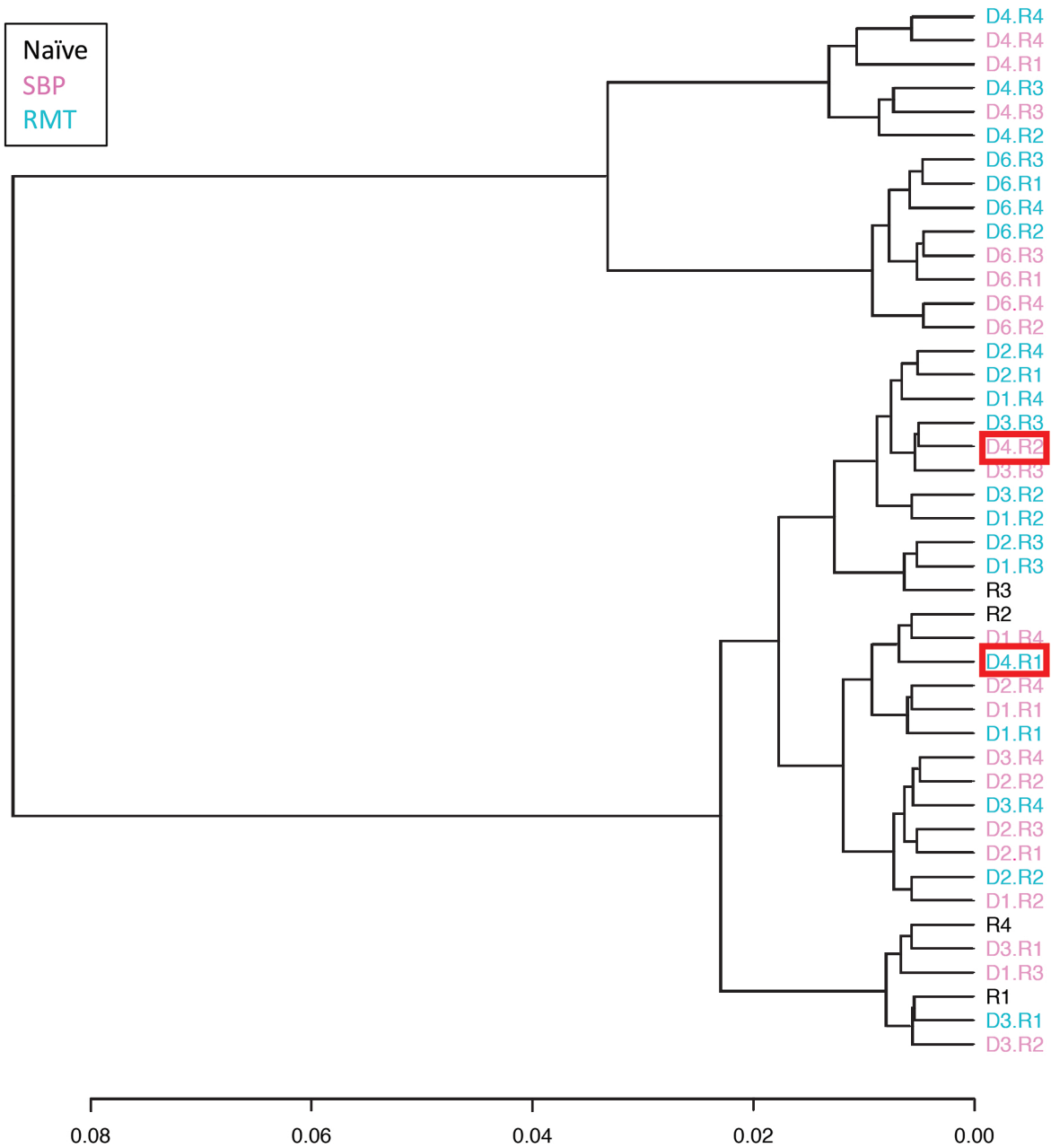

Supplement: Multimedia Component 1 — Supplementary Figure S1. Dendogram of sample correlation. Individual samples were organised into a dendogram to see how well the different samples correlated within a group. The red squares indicate the two samples that were removed from the analysis (RMT.R1 from D4 and SBP.R2 from D4) as they were found not to be infected. D, day of the blood-stage infection; R, replicate. [file mmc1.pdf]

A

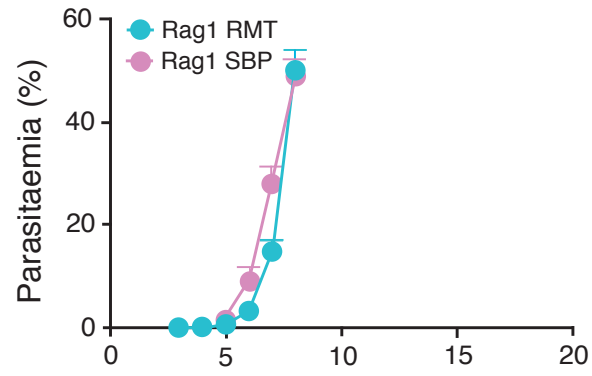

B

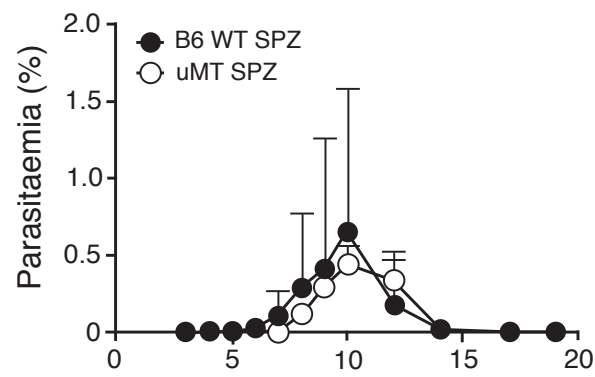

C

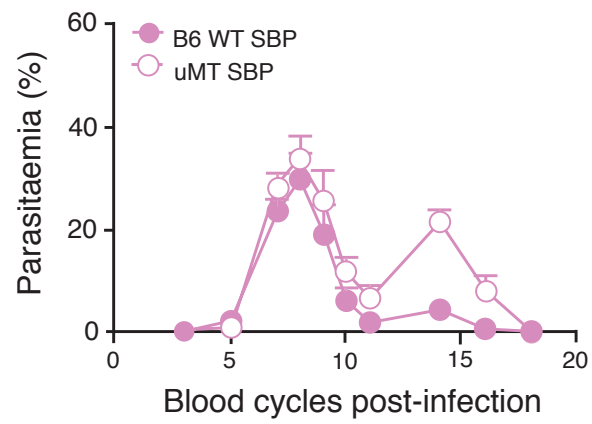

Supplement: Multimedia Component 2 — Supplementary Figure S2. The difference in parasitaemia is due to the immune response but B cells are not involved. Course of a P. chabaudi blood-stage infection in Rag1 knockout mice after intraperitoneal (i.p.) injection of 105 RMT-iRBCs (blue) or 105 SBP-iRBCs (pink) (A), μMT (open symbols) and wild-type C57Bl/6J mice (closed symbols) after intravenous (i.v.) injection of 100 sporozoites (SPZs) (B) or 105 SBP-iRBCs i.p. (C). The graphs show mean (+/- SEM) of percentage parasitaemia calculated from log-transformed data. Shown are data from one individual experiment with 8 mice/group (A) or 7 mice/group (B-C). [file mmc2.pdf]

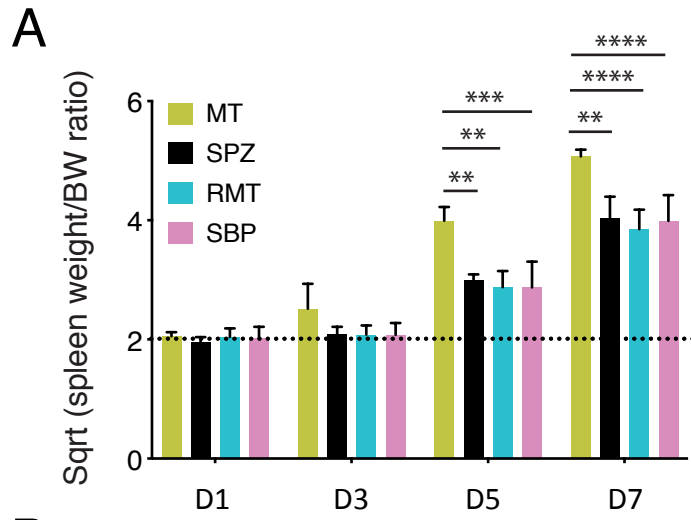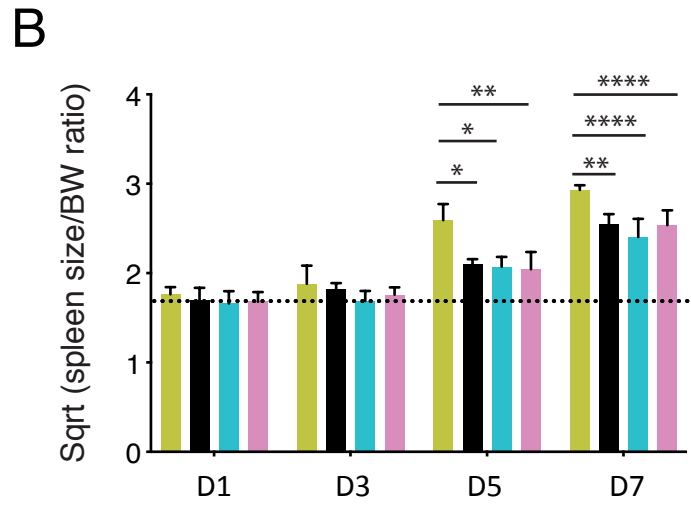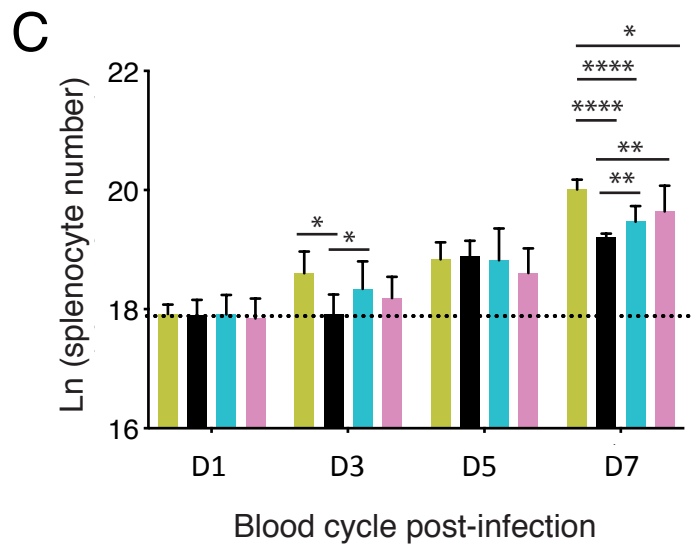

Supplement: Multimedia Component 3 — Supplementary Figure S3. No differences in the development of splenomegaly between RMT and SBP spleens in P. chabaudi blood-stage infections. Differences in splenomegaly between infection routes were investigated in three different ways: by comparing spleen weights (A), spleen sizes (length*width) (B), and splenocyte numbers (C) at different time points during the early infection phase from 7-10 weeks old C57Bl/6J mice infected by mosquito bite (MT) (green), i.v. injection of 100 SPZs (black), i.p. injection of 105 RMT-iRBCs (blue) or i.p. injection of 105 SBP-iRBCs (pink). Data were normalised by square root or log-transformation and spleen weight and size were normalised to the total body weight (BW) of the respective mouse from which the spleen was removed. Shown are pooled data from two experiments with 5-6 mice/group (MT), three to six experiments with 3-7 mice/group (RMT and SBP), or from one-three experiments with 5-6 mice/group (SPZ). Two-way ANOVA with Tukey's multiple comparisons test has been performed to test for differences between groups on a specific day. Lines above groups with asterisks illustrate significances between groups. *, p < 0.05; **, p < 0.01; ***, p < 0.001; ****, p < 0.0001. [file mmc3.pdf]

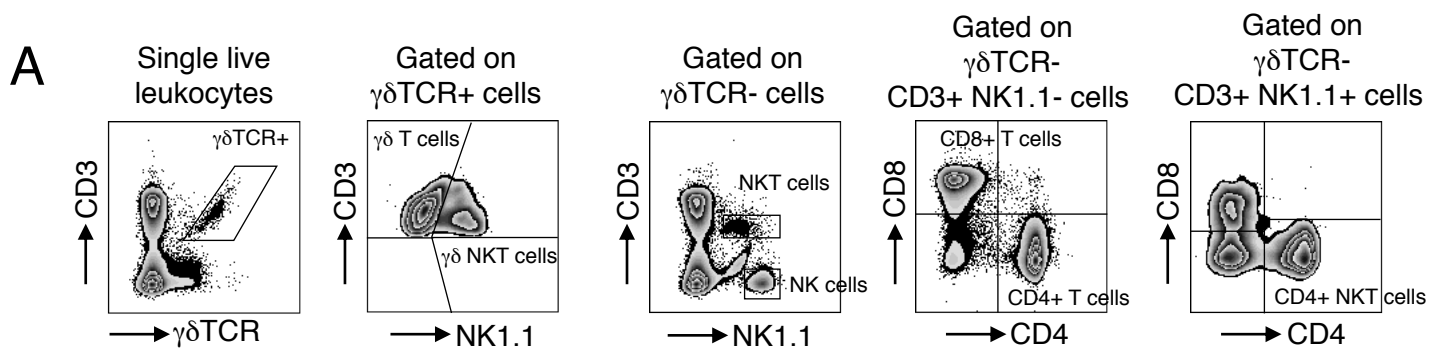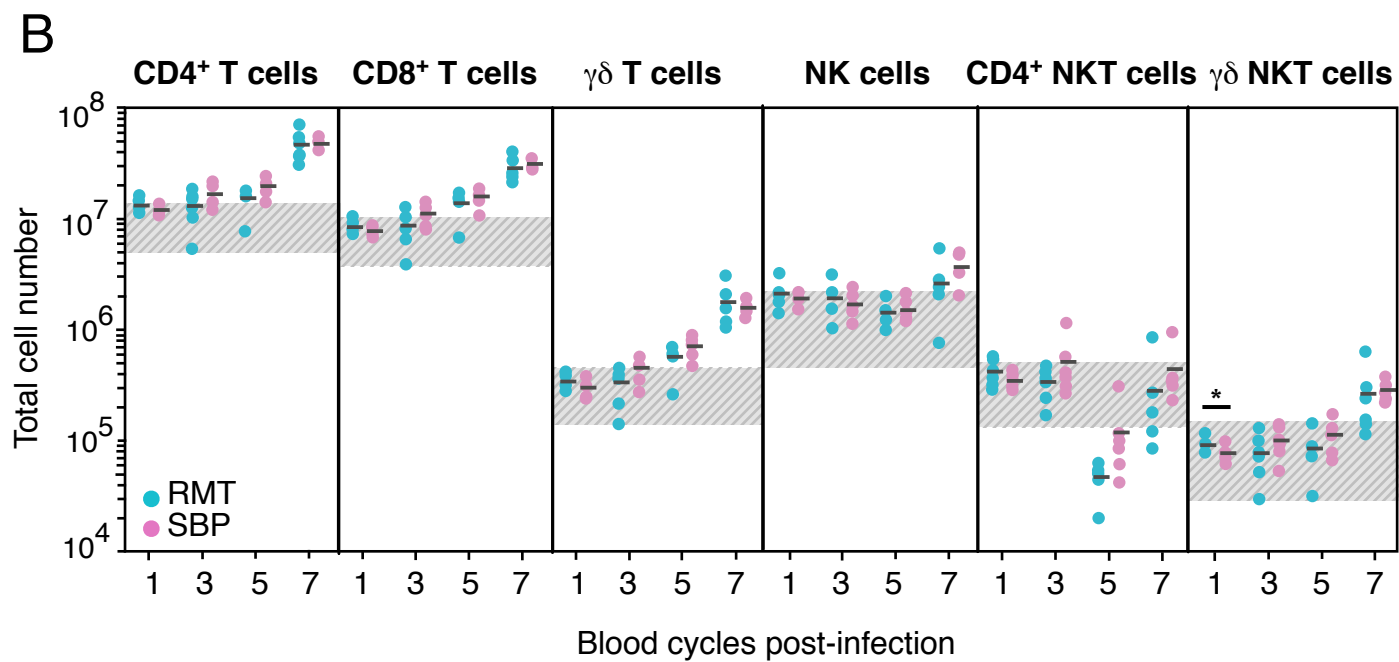

Supplement: Multimedia Component 4 — Supplementary Figure S4. No differences in total T and NK cell numbers in the spleen. C57Bl/6J mice were infected with 105 RMT-iRBCs or SBP-iRBCs or left uninfected. At the indicated time points after infection, mice were sacrificed, and their spleens removed and homogenised to obtain a single cell suspension. Cells were labelled with specific fluorescently-labelled antibodies as described in the materials and methods section and investigated by flow cytometry. A. Gating strategy for the investigated cell types. B. Total cell numbers of the indicated cell types in RMT (blue symbols), SBP (pink symbols) or uninfected control (grey dashed areas) spleens. Graph shows data from one individual experiment with 6 mice/group. Unpaired Mann-Whitney U tests were calculated to look for differences between RMT and SBP groups. Lines above groups with asterisks illustrate significances between groups. *, p < 0.05. NK(T) cells, natural killer (T) cells. [file mmc4.pdf]

B

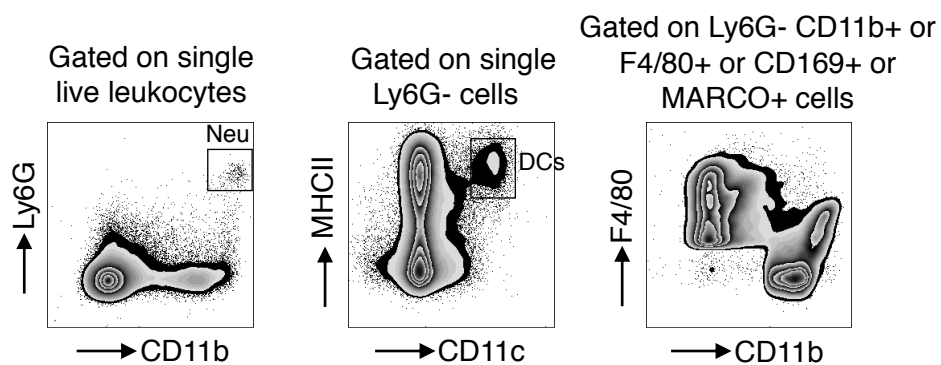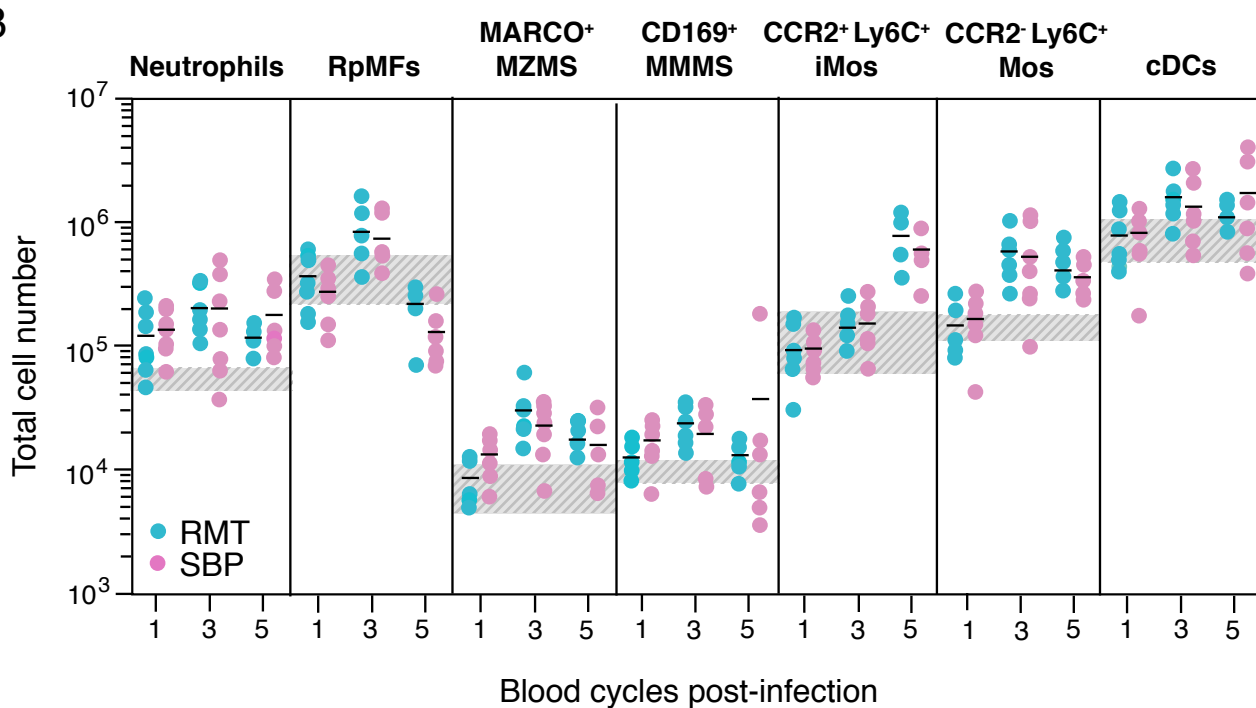

Supplement: Multimedia Component 5 — Supplementary Figure S5. No difference in splenic myeloid cells numbers. C57Bl/6J mice were infected with 105 RMT-iRBCs or SBP-iRBCs or left uninfected. At the indicated time points after infection, mice were sacrificed, and their spleens removed and homogenised to obtain a single cell suspension. Cells were labelled with specific fluorescently-labelled antibodies as described in the materials and methods section and investigated by flow cytometry. A. Gating strategy for the investigated cell types. B. Total cell numbers of the indicated cell types in RMT (bue symbols), SBP (pink symbols) or uninfected control (grey dashed areas) spleens. Graph shows data from one experiment with 6-7 mice/group. Unpaired Mann-Whitney U tests were calculated to look for differences between RMT and SBP groups but none of the comparisons were significant. RpMFs, red pulp macrophages; MZMs, marginal zone macrophages; MMMs, marginal metallophilic macrophages; iMos, inflammatory monocytes; Mos, monocytes; DCs, dendritic cells. [file mmc5.pdf]

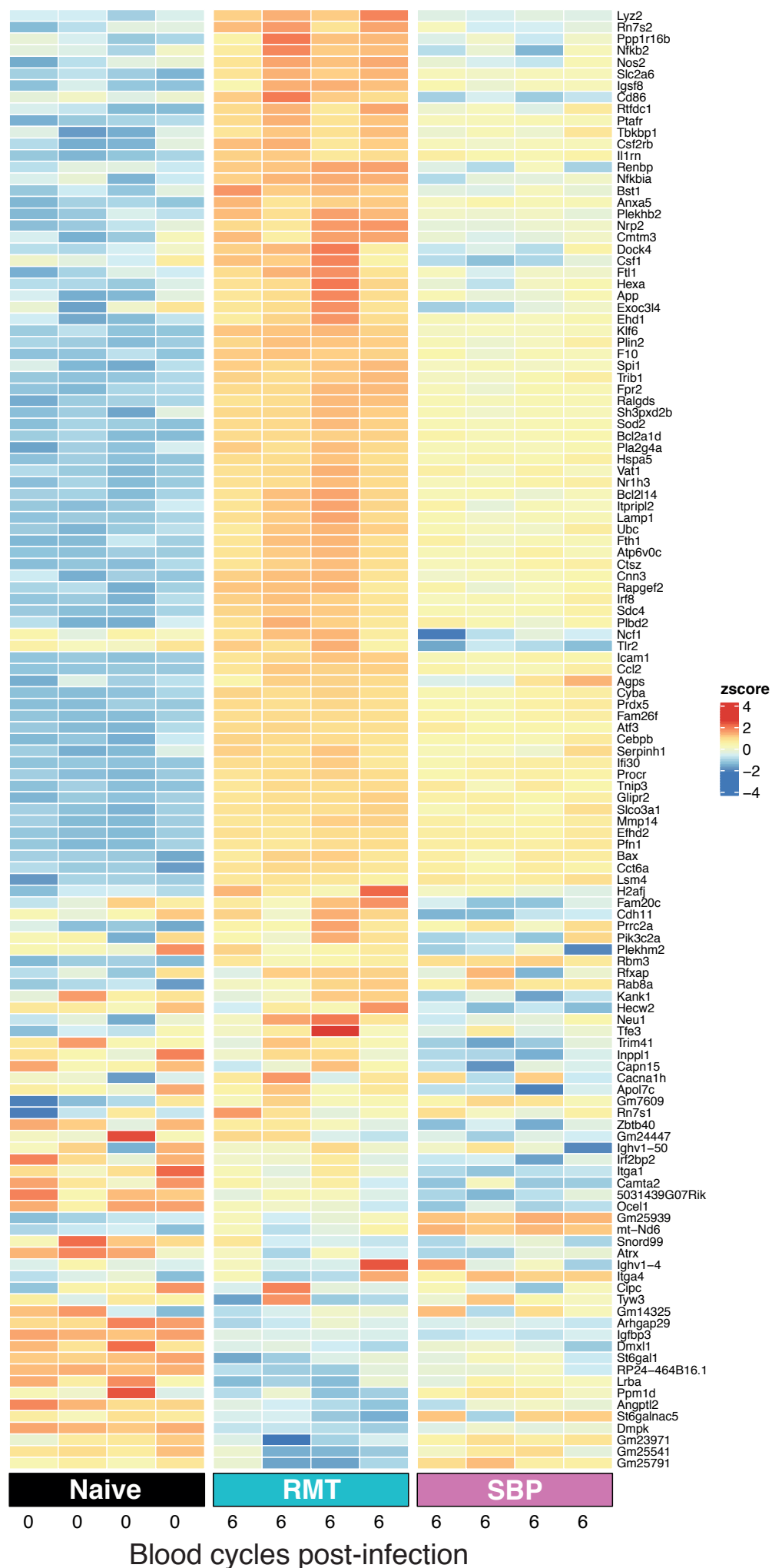

Supplement: Multimedia Component 6 — Supplementary Figure S6. Heatmap of unique RMT genes identified via the Likelihood Ratio Test. The Likelihood Ratio Test (LRT) was performed to look for changes in kinetics between RMT and SBP infections within the time series. The heatmap shows a number of genes that follow a unique pattern in RMT infections on day 6 of the infection in four uninfected control mice, four RMT infected mice and four SBP infected mice. Colours represent the Z-score values, which is a measurement of the number of standard deviations a sample value is above or below the mean across all samples for a given gene. [file mmc6.pdf]

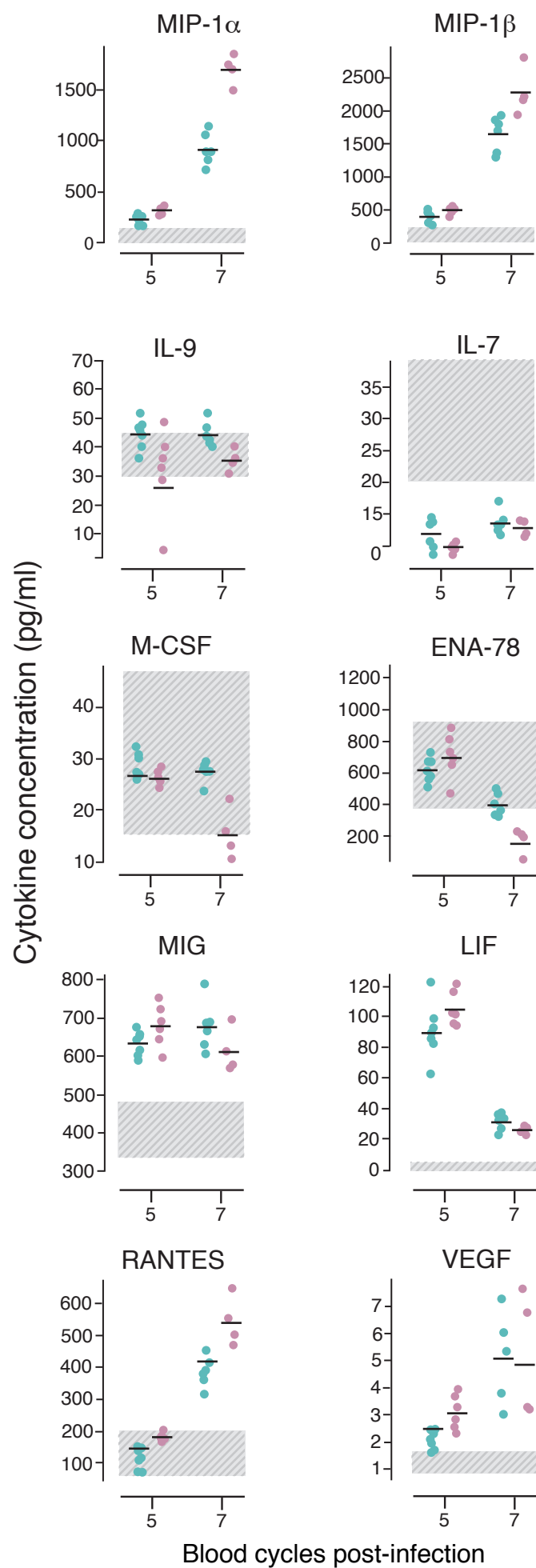

Supplement: Multimedia Component 7 — Supplementary Figure S7. Cytokines and chemokines in spleen lysates. Spleen lysates were prepared from C57Bl/6J mice infected with 105 RMT (blue symbols) or SBP-iRBCs (pink symbols) at different days post-infection (shown are day 5 and 7) or from naïve control mice (grey dashed areas) and investigated with a cytokine/chemokine protein array. Graphs depict protein content expressed as pg/ml for cytokines and chemokines that were not included in Figure 5. Graphs illustrate data from one experiment with 6 mice/group. The grey dashed areas denote the protein content of uninfected control samples. Transmission groups were compared per day via a nested ANOVA (see materials and methods section and Supplementary Table 5). [file mmc7.pdf]

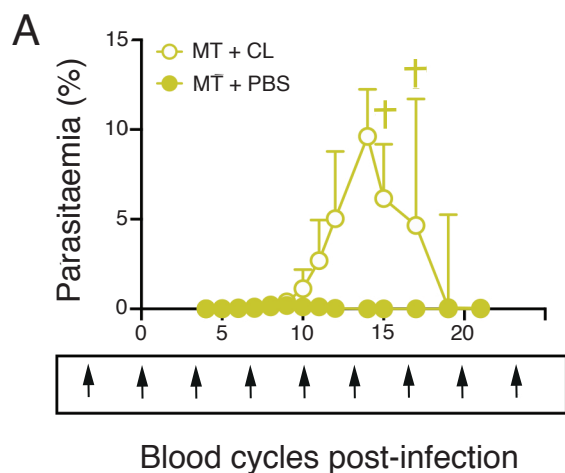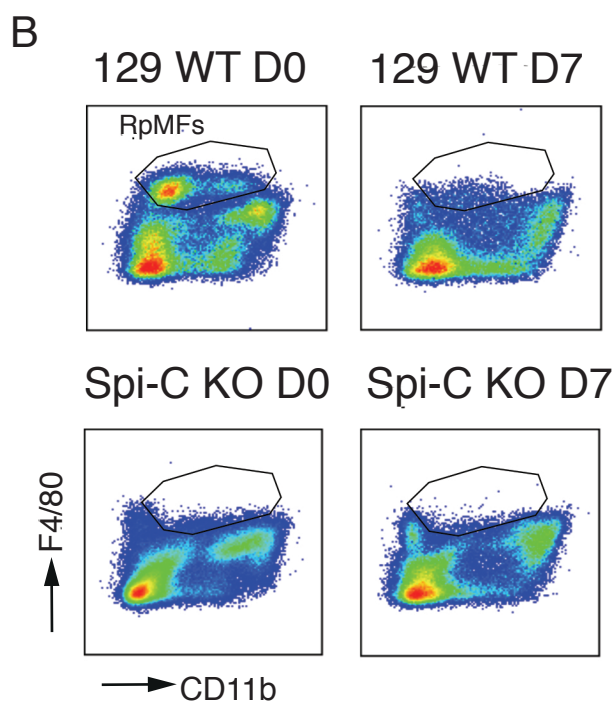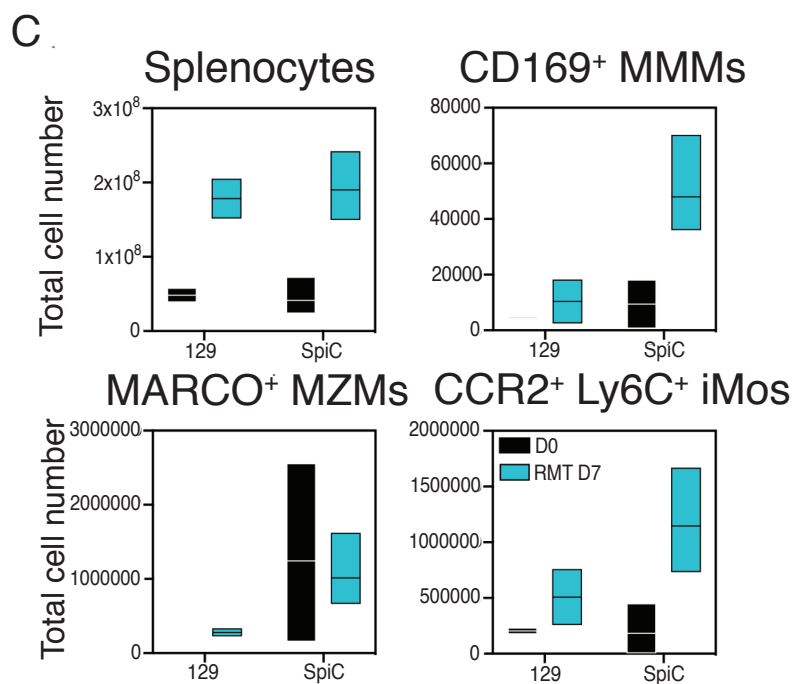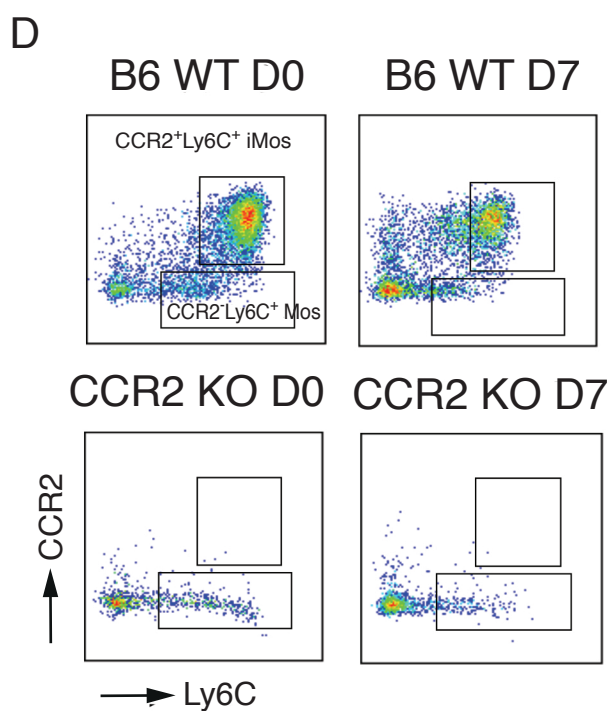

Supplement: Multimedia Component 9 — Supplementary Figure S9. Red pulp macrophages, but not recruited inflammatory monocytes, are important for attenuation of mosquito-transmitted P. chabaudi infections in C57Bl/6J mice. A. Course of a P. chabaudi blood-stage infection in C57Bl/6J mice treated with 200 uL clodronate liposomes (CL) i.v. (open symbols) or untreated control mice (closed symbols) infected via mosquito bite. Arrows underneath the graph illustrate time points of CL injection (first injection on day -4, i.e. two days before the bite, and after that every three days). B-D. Mice were infected with 105 RMT-iRBCs or left uninfected. At the indicated time points after infection, mice were sacrificed, and their spleens removed and homogenised to obtain a single cell suspension. Cells were labelled with specific fluorescently-labelled antibodies as described in the materials and methods section and investigated by flow cytometry. Panel B and D show the gating strategies for the investigated cell types in Spi-C knockout (KO) mice vs wild-type 129SvEv mice (B), and in CCR2 knockout (KO) mice vs C57Bl/6J wild-type (B6 WT) mice (D). Total cell numbers of the indicated cell types in RMT spleens on day 7 p.i. (D7, blue bars) or in uninfected control spleens (D0, black bars) are shown in panel C. The graph in panel A shows mean (+/- SEM) of percentage parasitaemia calculated from log-transformed data. Shown are data from one individual experiment with 9-13 mice/group. RpMFs, red pulp macrophages; MMMs, marginal metallophilic macrophages; MZMs, marginal zone macrophages; iMos, inflammatory monocytes; Mos, monocytes. [file mmc9.pdf]

A

Gated on single live leukocytes

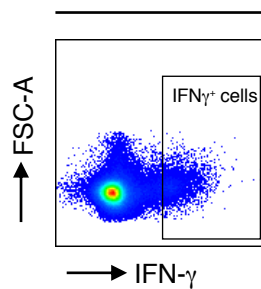Gated on IFN- $\gamma$ + cells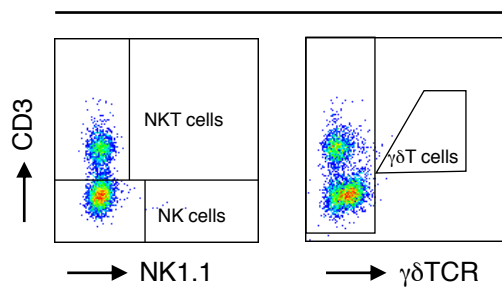

Gated on CD3+ NK1.1- cells

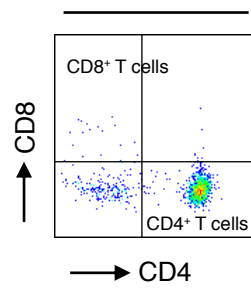

B

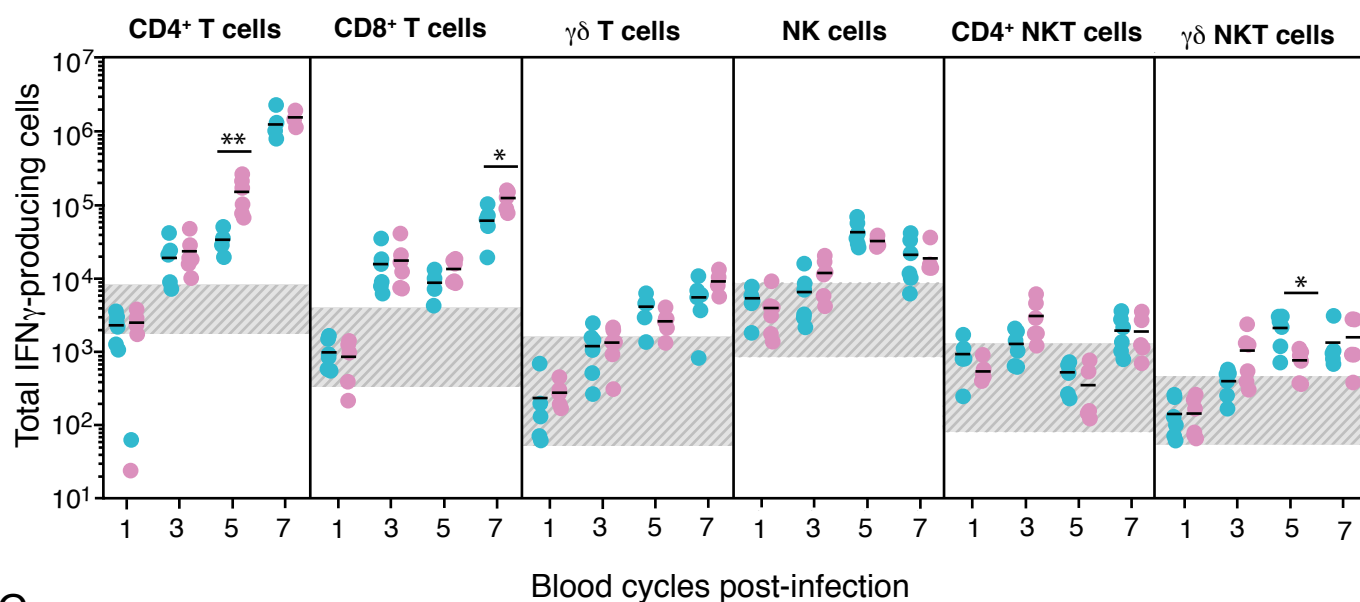

C

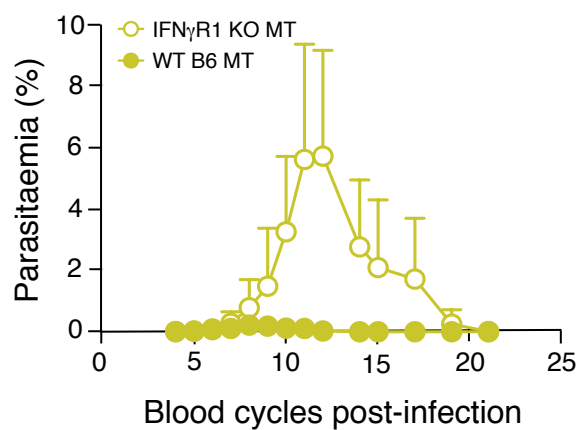

Supplement: Multimedia Component 10 — Supplementary Figure S10. No difference in the number of IFNγ-producing cells in the spleen. A-B. Mice were infected with 105 RMT-iRBCs or SBP-iRBCs or left uninfected. At the indicated time points after infection, mice were sacrificed, and their spleens removed and homogenised to obtain a single cell suspension. Cells were labelled with specific fluorescently-labelled antibodies as described in the materials and methods section and investigated by flow cytometry. (A) Gating strategy for the investigated cell types. (B) Total cell numbers of the indicated cell types in RMT (blue symbols), SBP (pink symbols) or uninfected control (grey dashed areas) spleens. Graph shows data from one experiment with 6 mice/group. Unpaired Mann-Whitney U tests were calculated to look for differences between RMT and SBP groups. Lines above groups with asterisks illustrate significances between groups. **, p < 0.01; *, p < 0.05. C. Course of a P. chabaudi blood-stage infection initiated via mosquito-bite in IFNγR1 knockout (KO) mice (open symbols) compared to wild-type C57Bl/6J (WT B6) mice (closed symbols). Graphs show mean (+/- SEM) of percentage parasitaemia calculated from log-transformed data. Shown are data from one experiment with 13 mice/group. [file mmc10.pdf]
