# Supplementary material for: Tissue macrophages and interferon-gamma signalling control blood-stage Plasmodium chabaudi infections derived from mosquito-transmitted parasites
Source: Curr Res Immunol. 2021 Jul 30;2:104–19. doi: 10.1016/j.crimmu.2021.07.002 (PMC8428512; doi:10.1016/j.crimmu.2021.07.002)
Supplement: Multimedia Component 8 — Supplementary Figure S8. Depletion of myeloid cells. C57Bl/6J mice were treated with clodronate (CL) or control (PBS) liposomes starting 2 days before infection with 105 RMT-iRBCs or SBP-iRBCs or left uninfected. Infected mice were treated with liposomes every 3 days. Mice were sacrificed at the day of infection (D0) after receiving one dose or 10 days post-infection (D10) after four doses of liposomes, and their spleens removed and homogenised to obtain a single cell suspension. Cells were labelled with specific fluorescently-labelled antibodies as described in the materials and methods section and investigated by flow cytometry. Panel A-B shows the gating strategy for the investigated cell types in control mice treated with one dose of control liposomes (PBS) (A) or clodronate liposomes (CL) (B) two days before. Panel C shows total cell numbers of the indicated cell types in RMT (blue bars), SBP (pink bars) or uninfected control (black bars) spleens. Graphs show data from one experiment with 4-5 mice/group (D0) and 3-8 mice/group for infected mice. Unpaired Mann-Whitney U tests were calculated to look for differences between PBS- and CL-treated groups. Asterisks denote significances compared to the PBS-treated group. *, p < 0.05; **, p < 0.01; ***, p < 0.001. Neu, neutrophils; DCs, dendritic cells; RpMFs, red pulp macrophages; MZMs, marginal zone macrophages; MMMs, marginal metallophilic macrophages; iMos, inflammatory monocytes; Mos, monocytes. [file mmc8.pdf]

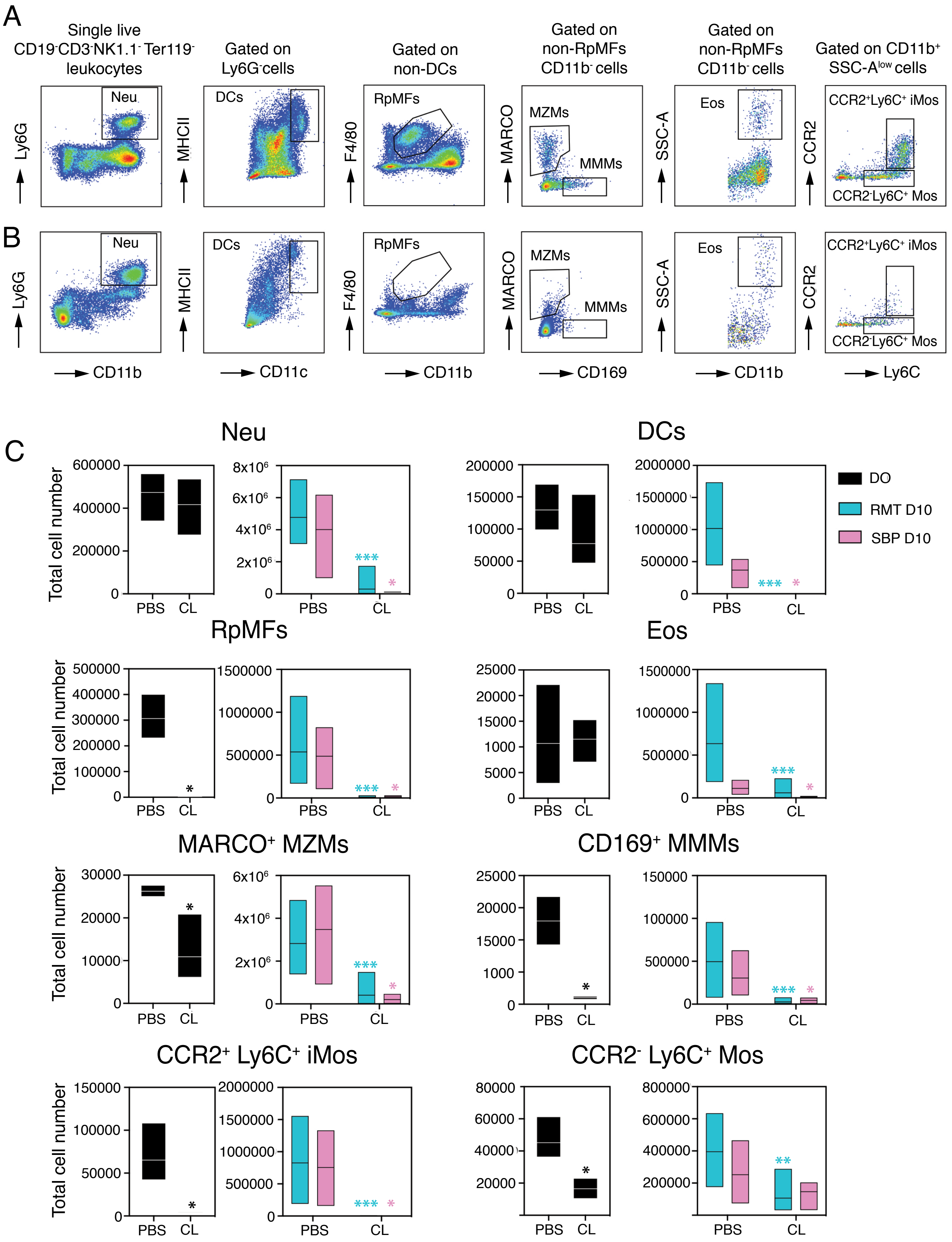
A
Single live CD19-CD3-NK1.1- Ter119- leukocytes
Gated on Ly6G-cells
Gated on non-DCs
Gated on non-RpMFs CD11b- cells
Gated on non-RpMFs CD11b- cells
Gated on CD11b+ SSC-Alow cells
Neu
DCs
RpMFs
MZMs
MMMs
Eos
CCR2+Ly6C+ iMos
CCR2-Ly6C+ Mos
Ly6G
MHCII
F4/80
MARCO
SSC-A
CCR2
B
CD11b
CD11c
CD11b
CD169
CD11b
Ly6C
C
Neu
DCs
RpMFs
Eos
MARCO+ MZMs
CD169+ MMMs
CCR2+ Ly6C+ iMos
CCR2- Ly6C+ Mos
Total cell number
PBS
CL
DO
RMT D10
SBP D10
***
**
*
